# Cryo‐EM Structures of Alcohol Oxidase Isozymes Reveal Structural Determinants of Cofactor Variation and Enzymatic Activity in *Ogataea methanolica*

**DOI:** 10.1111/1751-7915.70355

**Published:** 2026-04-18

**Authors:** Hao‐Liang Cai, Atsuhiro Shimada, Tasuku Hamaguchi, Akira Mizoguchi, Koji Yonekura, Kyohei Tsuchiyama, Masaya Shimada, Akio Ebihara, Kazutoshi Tani, Tomoyuki Nakagawa

**Affiliations:** ^1^ The United Graduate School of Agricultural Sciences Gifu University Gifu Japan; ^2^ The Graduate School of Natural Sciences and Technologies Gifu University Gifu Japan; ^3^ Faculty of Applied Biological Sciences Gifu University Gifu Japan; ^4^ RIKEN SPring‐8 Center Sayo Hyogo Japan; ^5^ Institute of Multidisciplinary Research for Advanced Materials Tohoku University Sendai Japan; ^6^ Graduate School of Medicine Mie University Tsu Mie Japan; ^7^ Preemptive Food Research Center Gifu University Gifu Japan; ^8^ Center for Computational Sciences University of Tsukuba Ibaraki Japan; ^9^ Center for Quantum and Information Life Sciences University of Tsukuba Tsukuba Ibaraki Japan

## Abstract

*Ogataea methanolica* is a methylotrophic yeast that can produce diverse recombinant proteins using methanol as the sole carbon and energy source. Unlike most yeast species, which possess a single alcohol oxidase, *O. methanolica* encodes two isoenzymes, Mod1p and Mod2p. This study examines the structural and functional differences between Mod1p and Mod2p homooctamers. Both enzymes were purified from *MOD*‐disrupted strains and analysed using cryogenic electron microscopy, achieving resolutions of 1.9 and 2.7 Å for Mod1p and Mod2p, respectively. The two isozymes assemble as tetramers of dimers stabilized by extensive intersubunit interactions, largely mediated by protruding loop regions and C‐terminal extensions. Despite overall structural similarities, Mod1p and Mod2p exhibit subtle differences in surface charge distribution and sequence composition within the FAD‐binding domain. These variations correlate with distinct cofactor preferences, with Mod1p binding arabityl FAD and Mod2p binding canonical FAD. Thin‐section electron microscopy further revealed that Mod1p and Mod2p form both homomeric and hybrid octamers that assemble into peroxisomal crystalloids essential for methanol metabolism. Collectively, our findings provide mechanistic insight into alcohol oxidase diversity in methylotrophic yeasts, advancing our understanding of methanol utilization and its applications in biotechnology.

## Graphical abstract

The methylotrophic yeast, *Ogataea methanolica* uniquely harbours two alcohol oxidase (AOD) isozymes, Mod1p and Mod2p, which exhibit distinct kinetic properties and are differentially regulated by methanol and oxygen availability, enabling metabolic adaptation to fluctuating environments. Here, we report high‐resolution cryogenic electron microscopy structures of the Mod1p and Mod2p homooctamers at 1.9 Å and 2.7 Å resolution, respectively.

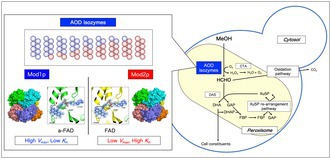

## 1 Introduction

Methanol is an attractive renewable carbon source that embodies the concept of a ‘methanol bio‐economy’ (Hartner and Glieder 2006; Fabarius et al. 2021). This is due to its status as a nonfood feedstock and a pure chemical compound as well as its ability to be completely utilized (Müller et al. 2015; Ochsner et al. 2015; Olah 2005; Pfeifenschneider et al. 2017). Methanol is a low‐impact carbon source. It can be synthesized from greenhouse gases, such as CO_2_ and methane (Blumberg et al. 2019; Hoppe et al. 2018). Therefore, microbial utilization of methanol can help establish a carbon‐neutral circulation system. The metabolic capabilities of methylotrophs, which can grow using methanol as the sole carbon and energy source, are crucial in realizing a ‘methanol based biorefinery’ (Fabarius et al. 2021; Cai, Wu, et al. 2022).

Methylotrophic yeasts, including *Komagataella phaffii* [formerly *Pichia pastoris* (Kurtzman 2005)], *Candida boidinii, Ogataea polymorpha* [formerly *Hansenula polymorpha* (Yamada et al. 1994)], *Ogataea minuta*, and *Ogataea methanolica* [formerly *Pichia methanolica* (Kurtzman and Robnett 1998)], are attractive hosts for realizing a *methanol based biorefinery*. This is because of the following reasons: (i) they can easily achieve high‐density cultures, (ii) they can be manipulated using many molecular genetic tools developed for them, and (iii) they possess robust methanol‐inducible promoters for gene expression (Hartner and Glieder 2006; Cos et al. 2006; Gellissen 2000; Raymond et al. 1998; Yurimoto and Sakai 2009; Yurimoto et al. 2011).

Since the identification of methylotrophic yeast more than 50 years ago (Ogata et al. 1969), significant progress has been made in elucidating the methanol‐utilizing pathway employed by methylotrophic yeasts. This pathway involves methanol oxidation and the xylulose monophosphate cycle for methanol assimilation (Rußmayer et al. 2015; Fukuoka et al. 2019). Alcohol oxidase (AOD: EC1.1.3.13) plays a pivotal role in this pathway (Ozimek et al. 2005) because of the following reasons: (i) AOD is present in all methylotrophic yeasts (Cregg et al. 1989; Ito et al. 2007), (ii) deletion mutants lacking AOD(s) cannot grow on methanol (Raymond et al. 1998; Cregg et al. 1989; Nakagawa et al. 1999), (iii) AOD catalyses the initial step of the pathway by oxidizing methanol into formaldehyde and hydrogen peroxide (van der Klei et al. 2006) and (iv) AOD is highly induced by methanol, accounting for ~30% of total cellular protein. This reaction takes place in the peroxisomes, which can occupy up to 80% of the cellular volume (Veenhuis et al. 1978).

Some methylotrophic yeasts, such as *O. methanolica*, possess nine isozymes of AOD, whereas most species typically have a single AOD (Ito et al. 2007; Cai, Shimada, and Nakagawa 2022). AOD isozymes are composed of subunits Mod1p and Mod2p, which are encoded by the *MOD1* and *MOD2*, respectively. Mod1p and Mod2p oligomerize randomly to form heterooligomers (Nakagawa et al. 1999, 2001; Cai, Shimada, and Nakagawa 2022). Although Mod1p and Mod2p share up to 85% amino acid sequence identity, they exhibit distinct enzymatic properties. Mod1p has a high *V*_max_ and low *K*_m_ against methanol and oxygen, whereas Mod2p has a low *V*_max_ and high *K*_m_ (Gruzman et al. 1996; Nakagawa et al. 1996, 2002). The FAD moiety in Mod1p is modified as arabityl flavin adenine dinucleotide (a‐FAD), and the proportion of a‐FAD is significantly higher in Mod1p than in Mod2p (Ashin and Trotsenko 1998). In *O. methanolica*, Mod1p is predominantly induced under low methanol and low oxygen conditions, enabling efficient assimilation of limited methanol (Cai, Shimada, and Nakagawa 2022; Nakagawa et al. 2002). By contrast, Mod2p is primarily expressed under high methanol and high oxygen conditions, thereby preventing accumulation of the toxic products formaldehyde and H_2_O_2_ (Cai, Shimada, and Nakagawa 2022; Nakagawa et al. 2002). This enables *O. methanolica* to adapt to changing environments by controlling methanol oxidation through AOD isozymes (Cai et al. 2021). However, the factors responsible for the differences in affinities for methanol and oxygen between Mod1p and Mod2p, are unknown at the molecular level.

The three‐dimensional (3D) structure of Aox1p, an AOD from *K. phaffii*, was determined using X‐ray crystallography and cryogenic electron microscopy (cryo‐EM) (Koch et al. 2016; Vonck et al. 2016). These works revealed the 3D structure of its active site, the binding domains for a‐FAD (rather than canonical FAD) and substrate, and the mechanism of catalysis. From quaternary structure analysis, Vonck et al. estimated the peroxisomal Aox1p crystal packing structure (Vonck et al. 2016; Vonck and van Bruggen 1992). This is relevant because AOD forms crystalloids in peroxisomes (Veenhuis et al. 1978). In vivo, AODs, as well as other methanol metabolic enzymes, such as dihydroxyacetone synthase and catalase, form crystalloids within the peroxisome matrix (Osumi et al. 1982). The AOD crystalloid structures facilitate methanol metabolism (Vonck and van Bruggen 1992). Therefore, the crystalloid structure of AOD isozymes within peroxisomes holds great importance in regulating methanol metabolism.

Recent advances in artificial intelligence–based structure prediction methods, such as AlphaFold3 (Abramson et al. 2024), have enabled many promising and highly accurate predictions for both monomeric and oligomeric protein structures. Although Aox1p shares more than 70% sequence identity with Mod1p and Mod2p, predicting structures containing more than 5000 residues is not currently feasible in a single AlphaFold3 run. The large total numbers of residues in the octameric Mod1p and Mod2p complexes (5312 and 5304 residues, respectively) therefore precluded structure prediction using this approach. Moreover, even if monomeric structures of Mod1p and Mod2p could be predicted, current structure prediction programmes are unable to infer their distinct cofactor‐binding preferences for a‐FAD or canonical FAD.

In this study, we present the 3D structure of homooctamers Mod1p and Mod2p from *O. methanolica* using cryo‐EM, with resolutions of 1.94 and 2.65 Å for Mod1p and Mod2p, respectively. Leveraging the structural information of both AOD subunits, we have elucidated the active site architecture, FAD‐ and substrate‐binding domains, and catalytic mechanisms of Mod1p and Mod2p in complex with a‐FAD and canonical FAD, respectively. Given the high structural similarity between Mod1p and Mod2p, we have inferred the presence of the 3D structure of hybrid octamers formed by Mod1p and Mod2p. To validate octamer assembly, we examined crystalloid structures of AOD isozymes in vivo in strains WT, *mod1*Δ and *mod2*Δ using thin‐section electron microscopy. Our findings provide insights into methanol metabolism in *O. methanolica*.

## 2 Materials and Methods

### 2.1 Yeast Strain and Culture Conditions

We used *O. methanolica* IAM 12901 (wild‐type), PMAD12 (Δ*mod1*) and PMAD13 (Δ*mod2*) (Raymond et al. 1998). YMP medium, containing 0.3% (w/v) yeast extract, 0.3% (w/v) malt extract, 0.5% (w/v) peptone and 0.01% (w/v) adenine, was used to induce AOD isozymes in *O. methanolica* strains. One of the following carbon sources was used for culturing the strains: 1% (v/v) methanol for strain Δ*mod2* and 3% (v/v) methanol for strain Δ*mod1*. The initial pH of the medium was adjusted to 6.2. The cells (100 mL) were cultured aerobically at 28°C with rotary shaking at 150 rpm in 500 mL Erlenmeyer flasks for 48 h.

### 2.2 Purification of Mod1p and Mod2p

Mod1p and Mod2p were purified as described (Nakagawa et al. 1996). Each protein showed a single band on standard SDS‐PAGE (Laemmli 1970) and was further assessed by negative‐stain EM using a JEM‐1010 instrument (Jeol Ltd., Tokyo, Japan).

### 2.3 Cryo‐EM Data Collection

Proteins for cryo‐EM were concentrated to 6.5 and ~4.4 mg/mL for Mod1p and Mod2p, respectively. In total, 3 μL of the protein solution was applied on a holey carbon grid (200 mesh Quantifoil R1.2/1.3 copper), which was Au‐sputtered and glow‐discharged for 10 s, blotted, and plunged into liquid ethane at −182°C using Vitrobot Mark IV (Thermo Fisher Scientific, Waltham, MA). The applied parameters were blotting time of 5 s with 10 blotting force at 100% humidity and 4°C. Data were collected at SPring‐8 on a CRYO‐ARM300 electron microscope (Jeol Ltd.) at 300 kV equipped with a K3 camera (Gatan Inc., Pleasanton, CA). An in‐column energy filter with a slit width of 20 eV was inserted to acquire movie frames. The movies were recorded using SerialEM (Mastronarde 2005) at a nominal magnification of 60,000 in counting mode. Pixel size was 0.823 Å at the specimen level. Dose rates were 14.47 and 13.18 e^−^ per physical pixel per second, corresponding to 21.37 and 19.47 e^−^ per Å^2^ per second at the specimen level for Mod1p and Mod2p, respectively. Exposure time was 2.0 s, resulting in an accumulated dose of 42.7 and 38.9 e^−^ per Å^2^ for Mod1p and Mod2p, respectively. Each movie included 50 fractioned frames.

### 2.4 Image Processing

The stacked frames were motion‐corrected using MotionCor2 (Zheng et al. 2017). Contrast transfer function (CTF) parameters was estimated using CTFFIND4 (Rohou and Grigorieff 2015). Particles picked using EMAN2 (Tang et al. 2007) were analysed with RELION 3.1 (Zivanov et al. 2018) and selected using 2D classification (Table S1 and Figures S2–S4). Initial 3D model was generated using EMAN2. The particles were divided into four classes using 3D classification, resulting in only one good class. Next, 3D autorefinement was performed with D4 symmetry for Mod1p and Mod2p following contrast transfer function refinement and Bayesian polishing. The relative orientations of the selected particles were iteratively refined to generate the final reconstructions. After masking and postprocessing, the final maps were obtained at resolutions of 1.94 and 2.65 Å for Mod1p and Mod2p, respectively, according to the gold‐standard Fourier shell correlation using a criterion of 0.143 (Figures S3 and S4 for Mod1p and Mod2p, respectively) (Rosenthal and Henderson 2003). Local resolution maps were calculated using RELION.

### 2.5 Model Building and Refinement of Mod1p and Mod2p Oligomers

Atomic model of the crystal structure of Aox1p from *Picha pastoris* (PDB: 5HSA) was fitted to the cryo‐EM map using Chimera (Pettersen et al. 2004). The model was manually adjusted and refined in real space using PHENIX (Adams et al. 2010), and the COOT (Emsley et al. 2010)/PHENIX refinement was iterated until the refinements converged. Following the refinement protocol for Aox1p (Koch et al. 2016), planarity restraints were removed from the pyrimidine ring of FAD, whereas planarity restraints were retained for dimethylbenzene and piperazine rings. Model quality was validated using MolProbity (Chen et al. 2010). Figures were prepared using PyMOL Molecular Graphic System (Schrödinger LLC, NY) and UCSF Chimera (Pettersen et al. 2004). Calculations for accessible surface area and buried interface area were performed using VMD (Humphrey et al. 1996). Sequence alignment was performed using ClustalW (Thompson et al. 1994) and visualized with ESPript 3.0 (Gouet et al. 2003).

### 2.6 Thermostability Assay

Differential scanning fluorimetry (DSF) experiments were performed as described previously (Niesen et al. 2007). Purified Mod1p and Mod2p were diluted to 10.8 μM in phosphate buffer (100 mM potassium phosphate, pH 7.0). The protein solutions were mixed with SYPRO Orange dye (final concentration 5×) in a 1:1 ratio, resulting in a final protein concentration of 5.4 μM. Fluorescence intensity was monitored using a StepOnePlus real‐time PCR instrument (Applied Biosystems, Foster City, USA) while the temperature was increased from 25°C to 95°C at a rate of 1°C/min. Fluorescence signals were recorded continuously as a function of temperature. The resulting thermal unfolding profiles were used to calculate the slope of the unfolding transition from the fluorescence curves using the calculation template (Niesen et al. 2007). The melting temperature (*T*_m_) was determined by fitting the fluorescence data to the Boltzmann equation using GraphPad Prism 11 (GraphPad Software, La Jolla, CA) and the calculation template (Niesen et al. 2007). Each measurement was performed in 10 replicates, and the Tm values were expressed as the mean ± standard deviation (SD).

Purified Mod1p and Mod2p were incubated at 40°C for 15, 30, and 60 min; these enzyme solutions were used for the AOD assay. AOD activity was determined using the ABTS/POD method, according to Tani et al. (1985). Three biological replicates were measured for each condition.

### 2.7 Ultrastructural Analysis

The cells were fixed using a rapid freezing or freeze‐substitution method. The samples were frozen in liquid propane at −175°C and freeze‐substituted with 2% glutaraldehyde, 1% tannic acid in ethanol, and 2% distilled water at −80°C for 2 days. The cells were stored at −20°C for 3 h, warmed up to 4°C for 4 h, dehydrated with ethanol three times for 30 min, and then dehydrated with ethanol at room temperature overnight.

The samples were infiltrated with propylene oxide (PO) two times for 30 min each; placed in a 50:50 mixture of PO and resin (Quetol‐651, Nisshin EM Co., Tokyo, Japan) for 3 h; and transferred to 100% resin overnight. After polymerization at 60°C for 48 h was complete, the resins were ultrathin sectioned at 70 nm with a diamond knife using an ultramicrotome (Ultracut UCT; Leica, Vienna, Austria). The sections were mounted on copper grids. The samples were stained with 2% uranyl acetate for 15 min, washed with distilled water, and secondary‐stained with lead stain solution (Sigma‐Aldrich Co., Tokyo, Japan) at room temperature for 3 min.

The grids were observed using a transmission electron microscope (JEM‐1400Plus, Jeol Ltd.) at an accelerating voltage of 100 kV with a CCD camera (EM‐14830RUBY2, Jeol Ltd.). *O. methanolica* strains were ultra highly analysed by Tokai‐EMA (Nagoya, Japan).

## 3 Results and Discussion

### 3.1 Overall Structures of the Mod1p and Mod2p Homooctamer

To elucidate the structural molecular mechanisms for Mod1p and Mod2p, each protein was subjected to cryo‐EM single particle analysis. Because both *MOD1* and *MOD2* are simultaneously induced by methanol in the *O. methanolica* wild‐type strain and are therefore difficult to separate using conventional purification methods, we purified Mod1p and Mod2p from *O. methanolica* Δ*mod2* and Δ*mod1* strains, respectively (see details in the Method section). SDS‐PAGE analysis revealed a single band for both Mod1p and Mod2p, with a size of ~67 kDa (Figure S1). Following cryo‐EM data collection and image processing using RELION, the final reconstructions achieved resolutions of 1.9 and 2.7 Å for Mod1p and Mod2p, respectively (Table S1 and Figures S2–S5).

**FIGURE 1:**
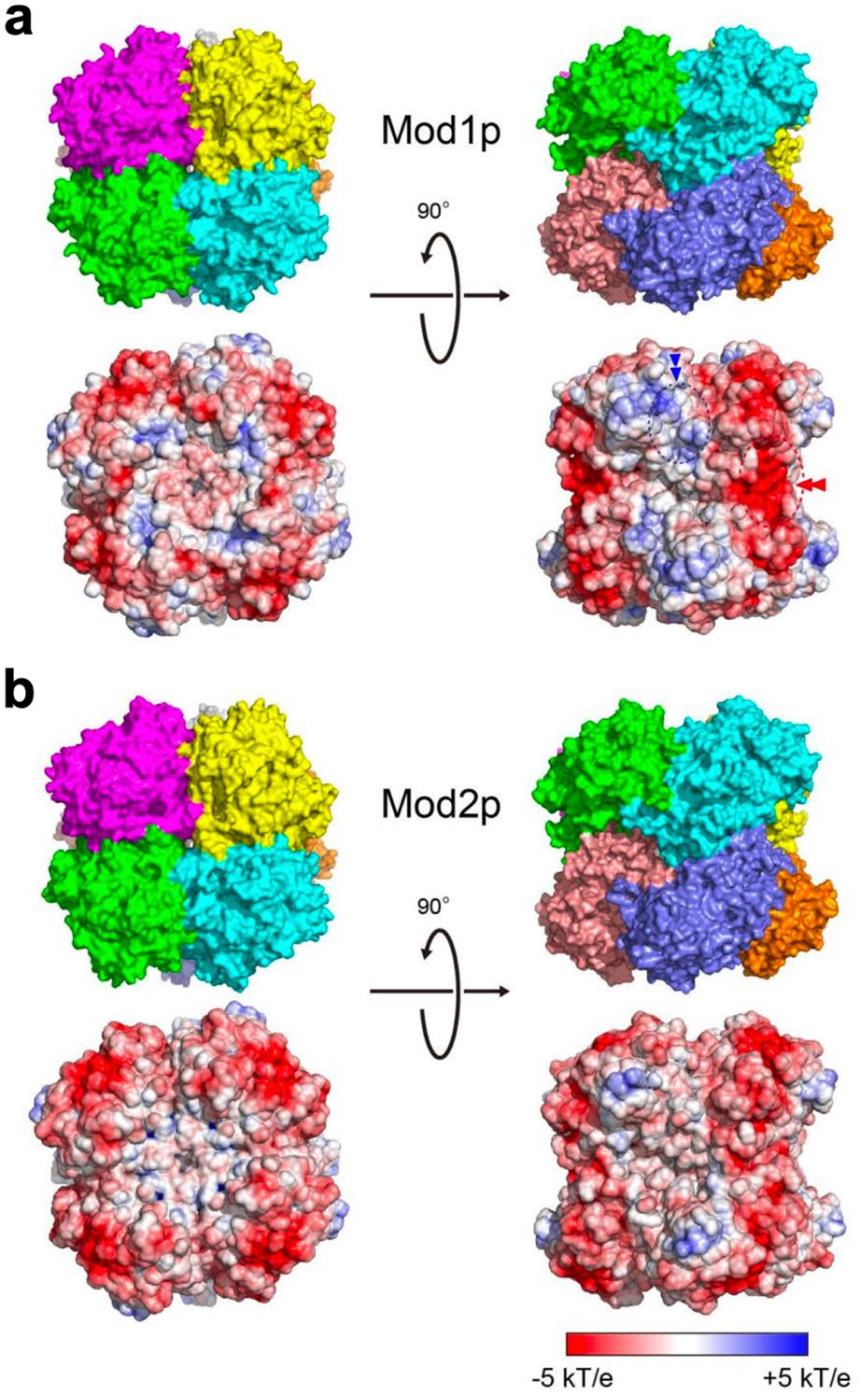
The octameric structures and surface charge distributions of *Ogataea methanolica* (a) Mod1p and (b) Mod2p. Each monomer is shown in a different colour. The top view is aligned along the fourfold axis, whereas the side view is obtained by rotating the top view by 90°. All charge distributions are colour‐coded according to the electrostatic potential ranging from −5 *kT/e* (red) to +5 *kT/e* (blue). Distinct charged regions in Mod1p are indicated by dashed ovals with arrowheads. The dashed ovals with blue and red arrowheads mark the region adjacent to the adenosine ring of FAD and the dimer interface formed by residues 484–561.

Similar to Aox1p from *K. phaffii* (Koch et al. 2016; Vonck et al. 2016) and vanillyl alcohol oxidase (VAO) from *Penicillium simplicissimum* (Ewing et al. 2020), *O. methanolica* Mod1p and Mod2p octamers assemble as a tetramer of dimers, which can be depicted as two tetramers docking in a face‐to‐face manner (Figure 1). In the tetramers of dimers, the buried dimer interface areas of Mod1p and Mod2p were 2942 and 2944 Å^2^, respectively (e.g., between the cyan and navy monomers). By contrast, the buried interfaces between the neighbouring dimers within the face‐to‐face docking tetramers were smaller, measuring 2027 Å^2^ for Mod1p and 1900 Å^2^ for Mod2p (93.8% of the Mod1p interface, e.g., between the green and cyan monomers). Based on these interface comparisons, the tetramer of dimers represents the most plausible minimal assembly unit. The nearly identical interface area of Mod1p and Mod2p suggests that they form heterodimers in vivo. Unlike the relatively weak dimer–dimer interactions in VAO, AOD complexes exhibit strong interactions between subunits. This stability is mainly conferred by two large protruding loop regions and an extended C‐terminal segment at the monomer periphery, which together reinforce the octameric assembly. Consequently, only a small active site cavity is observed at the centre, which may explain the preference of Aox1p, Mod1p and Mod2p for short chain alcohols, such as methanol, whereas VAO has broader substrate specificity for 4‐hydroxybenzylic compounds. Notably, differences in quaternary structures between Mod1p and Mod2p were nonsignificant.

**FIGURE 2:**
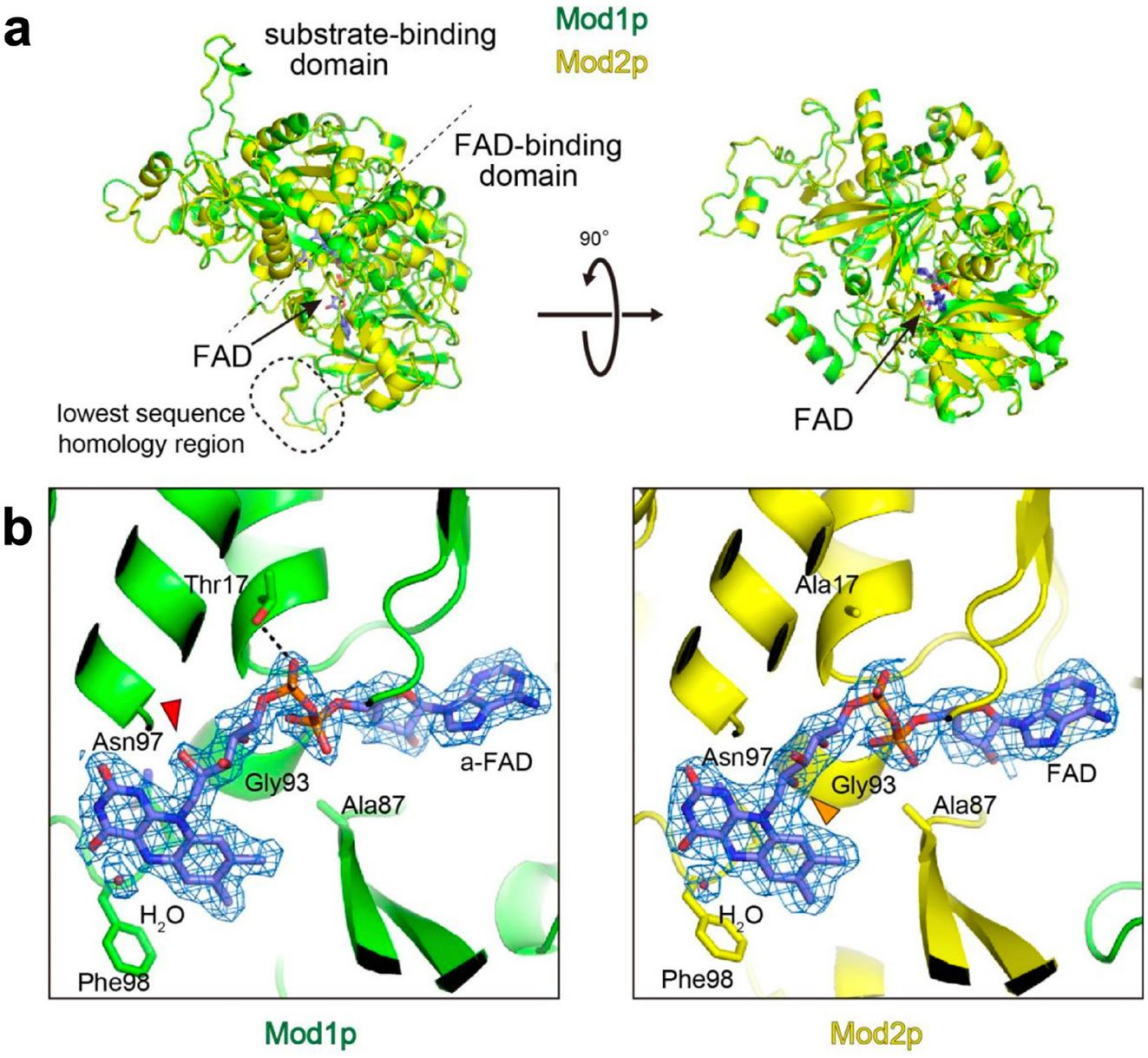
The structure of Mod1p and Mod2p monomers from *Ogataea methanolica*. (a) Top and side views of the Mod1p (green) and Mod2p (yellow) monomers, shown in cartoon representation with structural alignment. (b) FAD‐binding sites of Mod1p and Mod2p. The arabityl C2′‐OH group of a‐FAD of Mod1p is oriented towards the viewer (red arrowhead), whereas the C2′‐OH group of canonical FAD of Mod2p is oriented away from the viewer (orange arrowhead). A water molecule near the FAD isoalloxazine ring, presumed to be catalytically important, is shown. Dotted line indicates hydrogen bond. The density map is contoured at 4*σ*.

Consistent with those for members of the glucose–methanol–choline (GMC) family of oxidoreductases, high‐resolution structures showed that each subunit of Mod1p and Mod2p comprised an FAD‐binding domain (residues 1–155, 192–307 and 569–664 in Mod1p; residues 1–155, 192–306 and 568–663 in Mod2p) and a core substrate‐binding domain (residues 156–191 and 308–568 in Mod1p; residues 155–191 and 307–567 in Mod2p) (Figures 2a and Figure S6). The dimer and tetramer interfaces are primarily formed through the core substrate‐binding domain. The buried interface area of the Mod1p monomer is slightly larger than that of Mod2p, measuring 7025 and 6799 Å^2^ (96.8% of the Mod1p interface), respectively. This larger interface in Mod1p likely contributes to its enhanced assembly stability, consistent with the result of the enzymatic thermostability assay (Figure S7b). Notably, the C‐terminal extension (residues 654–664 in Mod1p and 653–663 in Mod2p), which includes peroxisomal targeting signal type 1 (PTS1) (residues 661–664 in Mod1p and 660–663 in Mod2p), is incorporated into the tetramer, similar to Aox1p, by associating with a neighbouring monomer. Most GMC oxidoreductases, which function as monomers or dimers, lack this C‐terminal extension.

These findings suggest that monomeric AOD subunits are transported into peroxisomes, because PTS1 is displayed on the surface of the subunits and Pex5p can recognize PTS1 on the subunits. By contrast, octameric AODs, which are assembled in the cytosol, are not transported into peroxisomes, because PTS1 is embedded within the octamer. This strongly supports the hypothesis that AODs are transported into peroxisomes as monomers and assembled into functionally active octamers in the peroxisome matrix (Stewart et al. 2001).

### 3.2 Differences in Surface Charge Distribution

The electrostatic potential map represents protein surfaces based on the Boltzmann equation, ranging from −5 *kT/e* (red) to +5 *kT/e* (blue), and was calculated using APBS (Baker et al. 2001). The negatively charged surfaces of Mod1p, Mod2p, and hybrid octamers are illustrated in Figure 1 and Figure S7, respectively. The charge distribution on the interaction surface between Mod1p and Mod2p shows minimal differences, reflecting their high sequence similarity (Figure S8). Variations in protein complex formation and structure are often determined by the number and strength of bonds between protein subunits (Szilágyi and Závodszky 2000; Kamiya and Shen 2003). Surface charge distribution also plays an important role in protein–environment interactions. In Mod1p, a more pronounced negatively charged region is present around a concave surface area, resulting from the substitution of Lys483 and Asn556 in Mod2p with His484 and Asp557 (red double arrowhead in Figure 1a). This substitution to His484, located near the oligomer dyad position, could contribute to the formation of a potential cation‐binding site and may therefore be associated with increased intersubunit interaction strength compared with Mod2p. In addition, Mod1p possesses a more positively charged region within a low‐homology segment adjacent to the adenine ring of FAD. Although direct experimental evidence for such an effect on FAD is currently lacking, electrostatics features near the isoalloxazine ring play an important role in modulating redox potential and electron transfer properties (Singh et al. 2025). Accordingly, the presence of two additional positively charged residues in this region, relative to Mod2p, may influence local loop conformation and thereby alter intersubunit associations, as discussed in a later section (blue double arrowhead in Figure 1a). Together with the negatively charged region described above, these complementary deviations in surface charge distribution are likely to contribute to oligomer stability and may also influence the guidance of methanol towards the enzyme surface through hydrogen bond formation (Noori et al. 2018).

### 3.3 Comparison of the Monomeric Structures Between Mod1p and Mod2p

**FIGURE 3:**
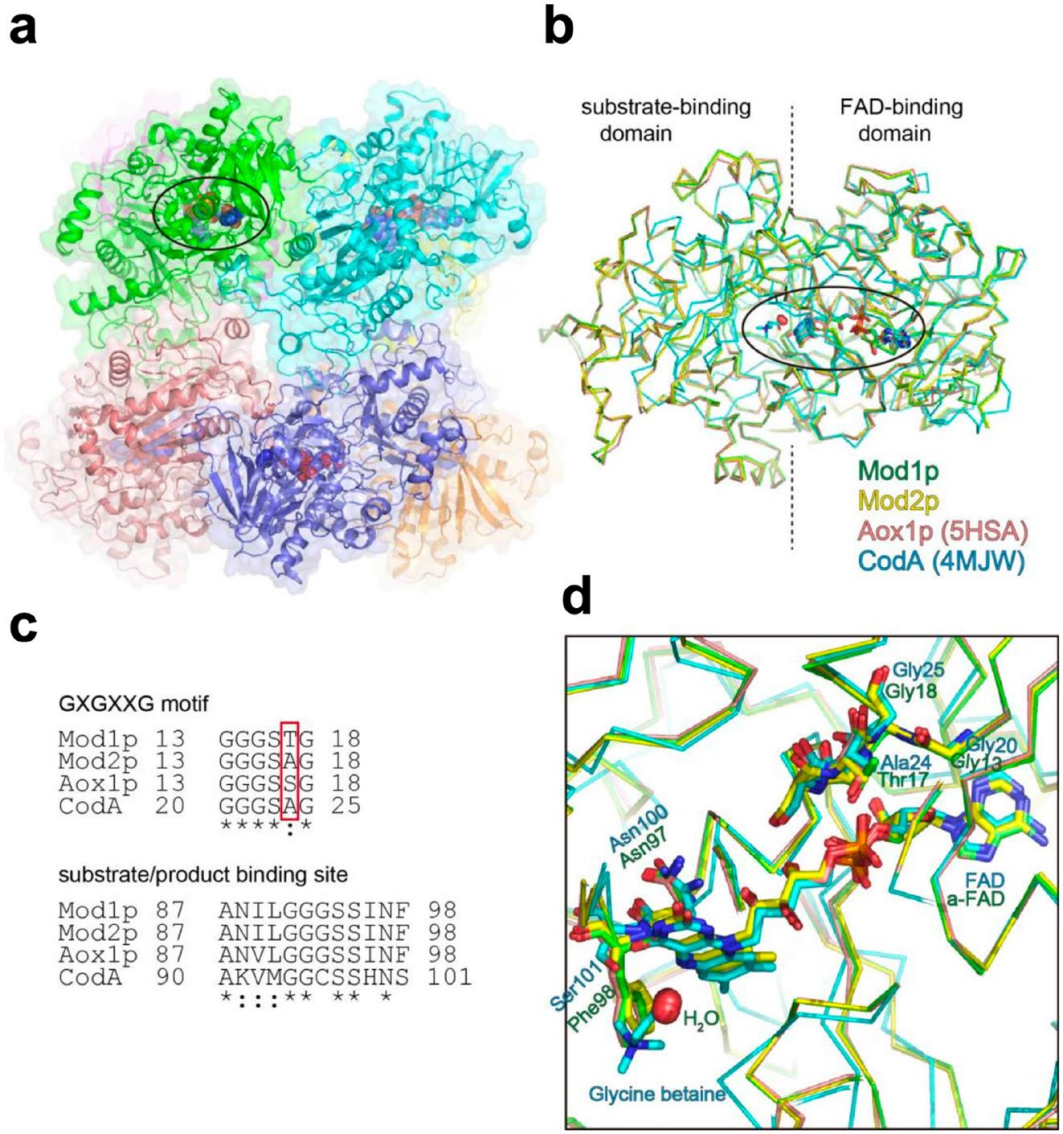
Structural comparison near the catalytically active site among GMC family members. (a) Surface and cartoon representation of the Mod1p octamer, with each monomer shown in a different colour, as in Figure 1. The FAD‐binding site is indicated by an oval. FAD molecules are depicted as sphere models. (b) Superposition of the Cα carbons of Mod1p (green), Mod2p (yellow), Aox1p (salmon; PDB: 5HSA), and choline oxidase CodA (cyan; PDB: 4MJW) is shown by ribbon representations. FAD and glycine betaine are depicted as stick models. Waters are depicted as spheres models. (c) Sequence alignment of the GXGXXG motif and substrate‐ or product‐binding sites of Mod1p, Mod2p, Aox1p and CodA was performed using ClustalW. The hydrophilic residues highlighted in the red box can form hydrogen bonds with the pyrophosphate group of FAD. (d) Magnified view of the FAD‐binding region in (b). Residues are labelled in green (Mod1p) and cyan (CodA).

Alignment of Mod1p with Mod2p monomers yielded a root mean square deviation of 0.272 Å for Cα position. Variations were observed at the periphery of the monomer, but structural differences at the octamer assembly site were nonsignificant (Figures 1 and 2). The FAD‐binding domain is the most conserved region, housing a noncovalently bound FAD molecule in the catalytic center—a characteristic shared with other GMC oxidoreductases (Figures 2b and 3). The flavin domain of FAD was coordinated by residues on the attachment loop, with Asn97 closely interacting with the flavin ring. The large side chain of Phe98 restricted the space available for the substrate. In addition, the backbone at Gly93 together with the side chain of Ala87 in both Mod1p and Mod2p contributed to narrowing the binding pocket surrounding the ribityl chain, compared with GMC oxidoreductases that oxidize larger alcohols. However, the bound FAD molecules adopted distinct forms in Mod1p and Mod2p, appearing as a‐FAD in Mod1p and canonical FAD in Mod2p (Figure 2b). In addition to their complexation with different FAD forms, the structural models of Mod1p and Mod2p monomers showed high similarity with each other and with Aox1p. The molecular basis for differential FAD‐binding is discussed in the following section.

### 3.4 Influence of Variable and Conserved Residues Around the Catalytic Site

As demonstrated by Koch et al. (2016), the substrate‐ or product‐binding site of AOD in methylotrophic yeast is adjacent to the FAD isoalloxazine ring, with a density peak corresponding to that of a water molecule (Figures 2b and 3d). This water molecule is presumed to be catalytically important, mimicking the position of the formaldehyde oxygen. The active sites and core substrate‐binding domains of Mod1p (green) and Mod2p (yellow) show a high degree of consistency. However, the substrate‐binding pocket differs for choline oxidase CodA from *Arthrobacter globiformis* complexed with glycine betaine (cyan; PDB ID: 4MJW) (Salvi et al. 2014). Specifically, the substrate‐binding pockets of Mod1p and Mod2p are smaller than that of CodA, due to the substitution of Ser101 in CodA with the bulkier Phe98 in Mod1p and Mod2p.

The replacement of Thr17 in Mod1p with Ala17 in Mod2p is noteworthy (Figure 2b and Figure S6). The hydroxyl group of Thr17 in Mod1p forms a hydrogen bond with the pyrophosphate moiety of FAD. The corresponding residue in *K. phaffii* Aox1p (Ser17) can form a similar hydrogen bond (Figures 2b and 3d). By contrast, the equivalent positions in Mod2p and CodA from *A. globiformis* are occupied by alanine (Ala17 and Ala24, respectively). Considering the different types of FAD bound by these enzymes, those containing threonine or serine at position 17, such as Mod1p and Aox1p, associate with a‐FAD, whereas those with alanine at the corresponding position, such as Mod2p and CodA, bind canonical FAD. Although the catalytic activity of FAD resides in the isoalloxazine ring, the stabilization of the adenine ring within the FAD‐binding domain likely contributes to molecular recognition. Thus, the presence of threonine or serine at position 17 may promote the binding of a‐FAD, playing an indirect yet functionally important role in modulating enzymatic activity. Furthermore, the exclusive occurrence of the modified a‐FAD moiety in Mod1p and Aox1p increases the possibility that these enzymes catalyse or facilitate autoepimerization, converting canonical FAD to a‐FAD (Bystrykh et al. 1991).

Phylogenetic analysis of partial amino acid sequences of AODs from methylotrophic yeast strains further supports the structural findings. AODs from four strains possessing two AOD genes—*Candida pignaliae* IAM 12906, *Candida sonorensis* IAM 12908, *O. methanolica* IAM 12901 and *Pichia* sp. BZ159—cluster within Group III (Ito et al. 2007). The second AODs from *O. methanolica* (*Om*Mod2p) and *C. sonorensis* (*Cs*Aod2p) form a subcluster in Group IIIB, and their FAD‐binding sequences are GGGSAG. By contrast, in Group IIIA, AODs have a conserved FAD‐binding sequence of GGGSTG. These structural and sequence differences, represented by Thr17 in Mod1p and Ala17 in Mod2p, particularly around the enzyme–substrate interaction site, likely underlie their distinct catalytic efficiencies towards methanol. Studies to clarify the mechanism are warranted.

### 3.5 Region With the Lowest Sequence Homology

**FIGURE 4:**
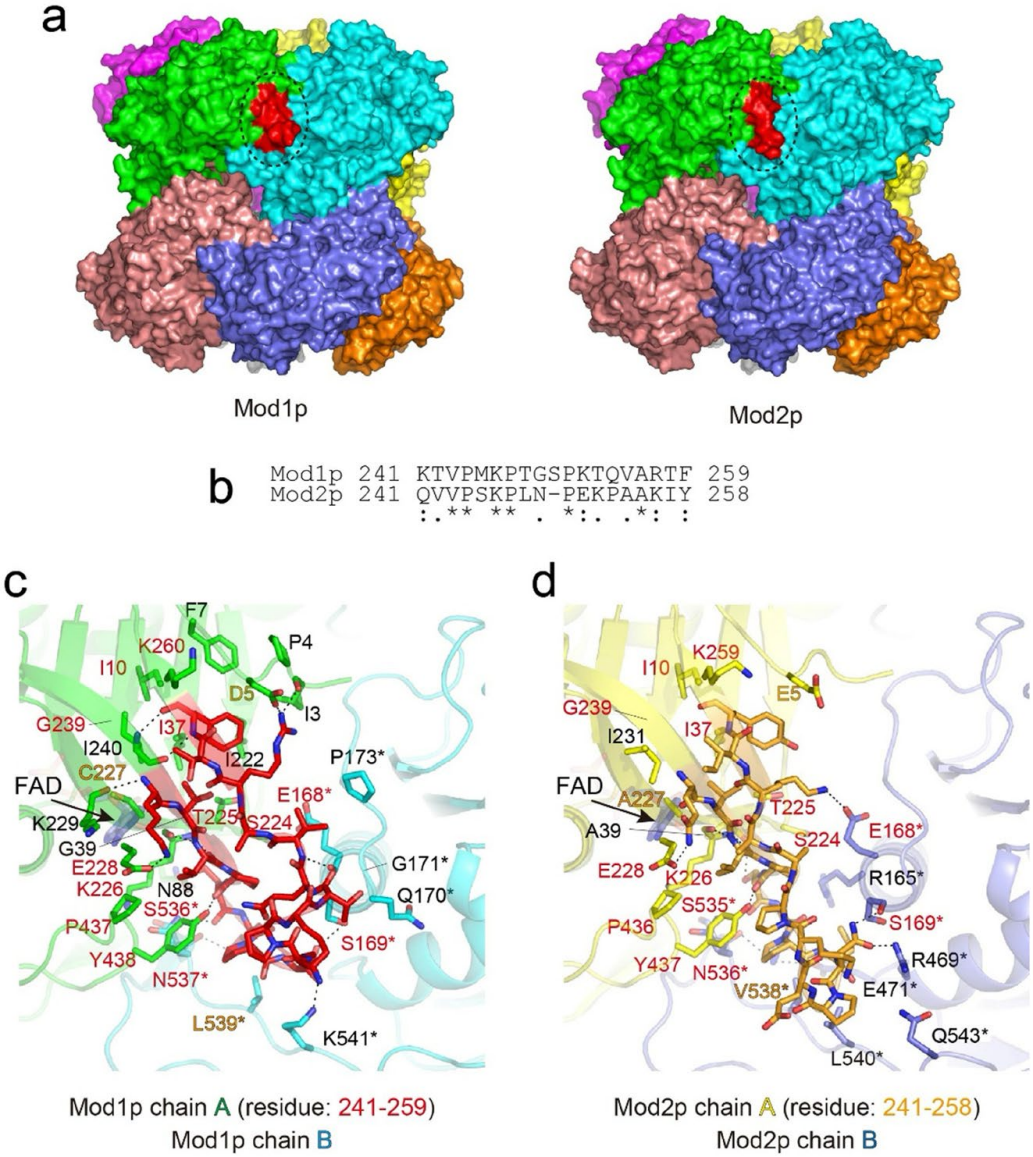
Region with the lowest sequence homology between Mod1p and Mod2p. (a) The surface models are colour‐coded as in Figure 1, excluding the lowest sequence homology region, highlighted in red (residues 241–259 in Mod1p and 241–258 in Mod2p). (b) Sequence alignment of the lowest sequence homology region between Mod1p and Mod2p was performed using ClustalW. (c, d) Magnified view of the region of Mod1p and Mod2p in (a), in the same orientation. Mod1p (green/cyan) and Mod2p (yellow/slate) monomers are depicted as cartoon representations. The lowest homology regions are highlighted in red for Mod1p and orange for Mod2p. Hydrogen bonds and salt bridges are indicated by dotted lines. Residue labels shown in red and orange denote positions that are identical or conserved interaction between Mod1p and Mod2p within the oligomer. Residues marked with an asterisk (*) indicate contribution from a neighbouring molecule.

Given that the amino acid sequence identity between Mod1p and Mod2p is up to 85%, we focused on a surfaced region (residues 241–259), which had the lowest homology in the FAD‐binding domain (Figure 4 and Figure S6). This peripheral region contains a loop (residues 245–252 in Mod1p and 245–251 in Mod2p) that interacts with neighbouring subunits. The intersubunit bond around the lowest region in Mod1p was tighter than that in Mod2p, indicating a more stable structure (Figure 4). Therefore, the molecular interface between the neighbouring dimers of Mod2p was reduced by ~6% compared with that of Mod1p, as described in the section *Overall structures of the Mod1p and Mod2p homooctamer*.

To characterize this lowest sequence homology region, we examine the thermal stability of the Mod proteins using differential scanning fluorimetry (DSF). This method monitors protein unfolding in the presence of a hydrophobic fluorescent dye, with fluorescence intensity recorded as a function of temperature (Niesen et al. 2007). The DSF profiles (Figure S7a) showed that each Mod protein exhibited an approximately two‐state transition during thermal denaturation. The *T*_m_ values of Mod1p and Mod2p were 50.8°C ± 0.24°C and 52.0°C ± 0.11°C, respectively, indicating comparable overall thermal stabilities. The onset temperatures of thermal unfolding were 34.7°C ± 1.1°C for Mod1p and 39.0°C ± 0.6°C for Mod2p, suggesting that thermal Mod1p begins to unfold at a slightly lower temperature. The slope values of the unfolding transition for Mod1p and Mod2p were 5.38 ± 0.29 and 9.2 ± 0.20(/°C), respectively, indicating that once unfolding is initiated, the transition of Mod2p proceeds more steeply than that of Mod1p.

Both Mod1p and Mod2p form octameric assemblies. In principle, thermal denaturation of oligomeric proteins can involve multiple transitions corresponding to oligomer dissociation followed by subunit unfolding. However, the DSF profiles of Mod1p and Mod2p exhibited a single, approximately two‐state transition. This observation suggests that thermal unfolding does not proceed through clearly separated dissociation steps, such as octamer‐to‐tetramer conversion. Instead, the unfolding process appears to occur in a largely cooperative manner without detectable intermediate oligomeric states, possibly reflecting progressive destabilization of individual subunits within the octamer.

Although the Tm values determined by DSF were similar for Mod1p and Mod2p, residual enzyme activity measurements showed that Mod1p retains higher catalytic activity after incubation at 40°C (Figure S7b). This discrepancy suggests that thermal inactivation may result from local structural perturbations affecting the active site rather than from global protein unfolding. Consistent with the nearly identical oligomeric interfaces of Mod1p and Mod2p, the low‐homology region is located near both the FAD‐binding site and the oligomer interface (Figure 4). This region appears to provide a structurally and functionally diverse element within the oligomeric assembly, contributing to differences in enzymatic thermal stability and oligomer stabilization between Mod1p and Mod2p (Figure S7). Therefore, this low‐homology region may play an important regulatory role in modulating methanol metabolism.

### 3.6 Formation of Crystalloids in Peroxisomes

In vivo, AODs form crystalloids with other methanol metabolizing enzymes, such as dihydroxyacetone synthase and catalase, within the peroxisome matrix—a structural arrangement for efficient methanol metabolism (Vonck and van Bruggen 1992). Although single AODs in *C. boidinii* and *O. angsuta* formed crystalloids in peroxisomes (van der Klei et al. 2006; Veenhuis et al. 1978, 1981; Osumi et al. 1982), evidence lacks in establishing whether AOD isozymes, consisting of two homooctamers and seven hybrid octamers, form crystalloids in peroxisomes. The alignment of alcohol oxidases forming crystalloids in peroxisomes highlights the conservation of residues implicated in crystal contact regions around Helix 12 (H12) (Lys287/286 and Arg290/289 in Mod1p/Mod2p), consistent with peroxisomal crystal packing inferred from the Aox1p structure of *K. pastoris* (Vonck et al. 2016) (Figures S6 and S9). Helix 16, which contains two conserved glutamate residues (Glu368/367–Glu369/368 in Mod1p/Mod2p), appears to be spatially distant from H12 and is therefore unlikely to be directly involved; however, it may contribute to proper crystalloid organization due to its position between adjacent octamers. Thus, it is plausible that heterooctamers form crystalloids.

**FIGURE 5:**
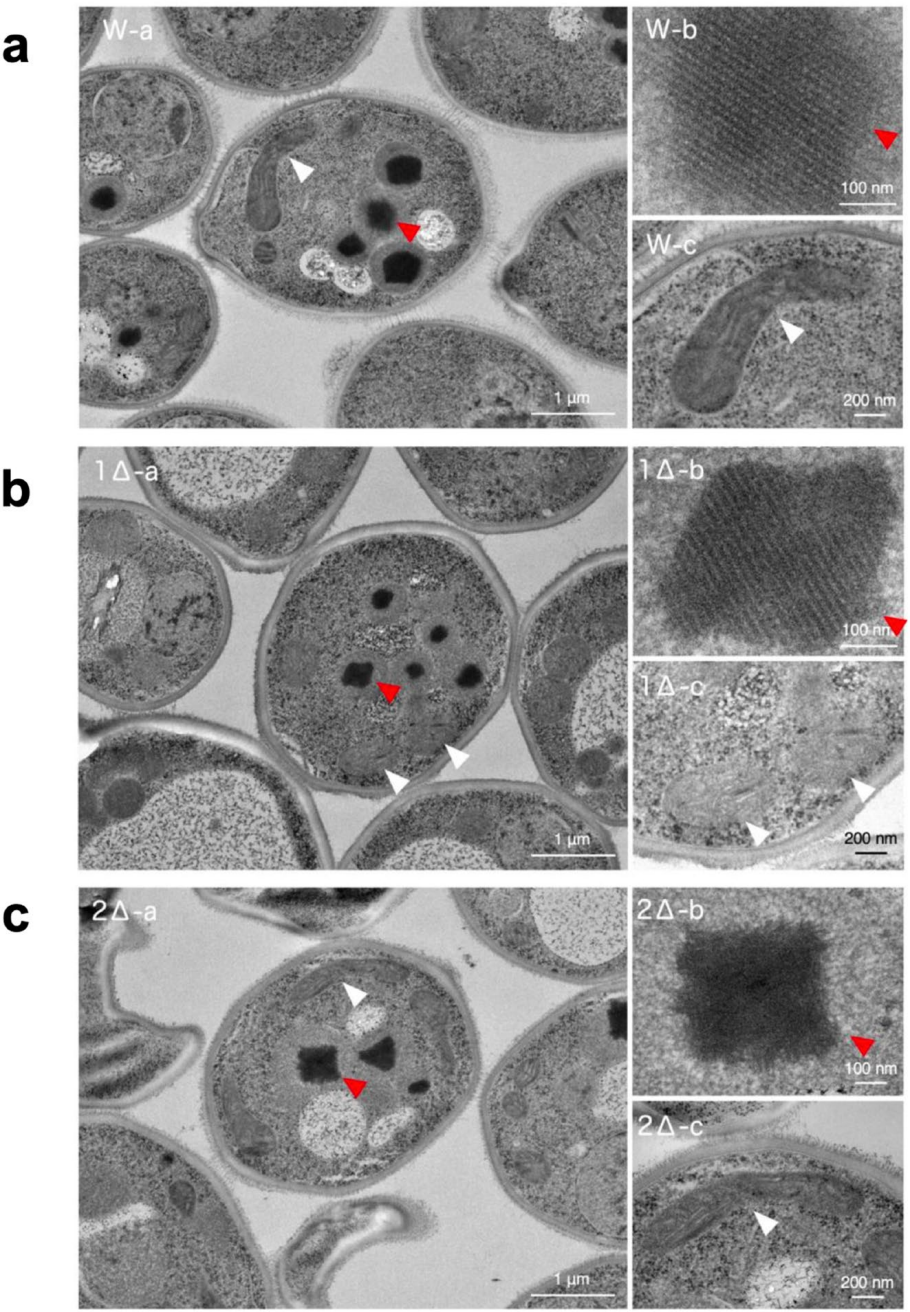
Subcellular structure and morphology of *Ogataea methanolica* observed using thin‐section electron microscopy. Morphology of cells (−a), peroxisomal crystalloids (−b) and mitochondria (−c) in *O. methanolica* WT (a), Δ*mod1* (b), and Δ*mod2* (c). White and red arrowheads indicate mitochondria and crystalloids, respectively.

To test this hypothesis, we used thin‐section electron microscopy to examine the subcellular structure and morphology of *O. methanolica* grown on methanol. Similar to that observed for *C. boidinii* and *O. angusta* , homooctamers of Mod1p and Mod2p, synthesized in strains Δ*mod2* and Δ*mod1*, respectively, formed crystalloids in peroxisomes (Figure 5). Notably, when the wild‐type *O. methanolica* strain IAM12901 was grown on methanol, it formed crystalloids in peroxisomes, indicating that Mod1p and Mod2p together form crystalloids. Although we speculate on conserved potential interaction sites within homooligomeric crystalloids based on the Aox1p structure of *K. pastoris* (Vonck et al. 2016), Figure 5 does not provide sufficient structural detail to definitively characterize these interactions. Further investigation is required to reveal the peroxisomal crystal packing structure of AOD isozymes using the 3D structure of Mod1p and Mod2p. Such molecular information would be valuable for understanding the mechanisms of methanol metabolism in *O. methanolica*.

## 4 Conclusion

In this study, we present high‐resolution cryo‐EM structures of both alcohol oxidase isoforms, Mod1p and Mod2p, from *Ogataea methanolica*, providing detailed structural insights into their architecture and functional diversity. Despite their high overall structural similarity, our analyses reveal key differences in cofactor binding, with Mod1p accommodating a‐FAD and Mod2p binding canonical FAD. These findings highlight subtle but important variations within the FAD‐binding domain that are not currently captured by structure prediction methods such as AlphaFold3 (Abramson et al. 2024). In addition to cofactor specificity, our structural and biochemical analyses identify regions that may contribute to differences in enzymatic properties and oligomer stability between the two isoforms. In particular, variations near the catalytic site and at intersubunit interfaces suggest possible mechanisms underlying their distinct catalytic behaviours and stability profiles. The high degree of structural conservation, including the low RMSD and similar oligomeric interfaces, further supports the potential for heterooligomer formation, consistent with previous zymogram analyses demonstrating hybrid oligomers in vivo and in vitro (Nakagawa et al. 1996).

More broadly, the structures reported here provide valuable experimental benchmarks for improving AI‐based protein structure prediction, particularly for large oligomeric complexes and cofactor‐dependent enzymes. Although the precise molecular basis underlying the differences in catalytic efficiency between Mod1p and Mod2p remains to be elucidated, our findings establish a structural framework for future investigations, including targeted mutagenesis and studies of peroxisomal assembly and crystalloid formation. Overall, this work clarifies the structural determinants underlying alcohol oxidase diversity in methylotrophic yeast and highlights its relevance to methanol metabolism, with potential implications for biotechnology and synthetic biology applications.

## Author Contributions

**Hao‐Liang Cai:** writing – original draft, writing – review and editing, investigation, data curation. **Akio Ebihara:** writing – review and editing, investigation, data curation. **Masaya Shimada:** data curation, investigation, writing – review and editing. **Kazutoshi Tani:** conceptualization, investigation, methodology, data curation, supervision, project administration, funding acquisition, writing – original draft, writing – review and editing. **Akira Mizoguchi:** data curation, investigation, writing – review and editing. **Tomoyuki Nakagawa:** supervision, conceptualization, project administration, funding acquisition, writing – original draft, writing – review and editing. **Atsuhiro Shimada:** methodology, data curation, investigation, writing – review and editing. **Koji Yonekura:** writing – review and editing, investigation, data curation. **Tasuku Hamaguchi:** writing – review and editing, investigation, data curation. **Kyohei Tsuchiyama:** data curation, investigation, writing – review and editing.

## Funding

This research was partially supported by the Platform Project for Supporting Drug Discovery and Life Science Research (Basis for Supporting Innovative Drug Discovery and Life Science Research) from AMED (Grant Numbers; JP21am0101118, JP22ama121006 and JP25ama121004), and JST‐Mirai Program2 (Grant Number JPMJMI23G2). This work is also partly supported by a KAKENHI, Grant‐in‐Aid for Challenging Exploratory Research (18K19875) to T.N. from the Japan Society for the Promotion of Science (JSPS). This work is partly supported by Center for Quantum and Information Life Sciences, University of Tsukuba.

## Conflicts of Interest

The authors declare no conflicts of interest.

## Supporting information


**Figure S1:** SDS‐PAGE of purified Mod1p and Mod2p. A single band of Mod1p and Mod2p showing the size of around 67 kDa.
**Figure S2:** Cryo‐EM micrographs of the Mod1p and Mod2p octamer. A representative cryo‐EM (a) and (c) micrograph of purified Mod1p and Mod2p particles, respectively. (b) and (d) indicate representative 2D class averages from cryo‐EM micrographs of Mod1p and Mod2p, respectively.
**Figure S3:** Structure determination of Mod1p by cryo‐EM. (a) Image processing flow of 3D classification and reconstruction. (b) Fourier shell correlation (FSC) plots of the cryo‐EM map (unmasked: black, masked: blue, phase‐randomized corrected: green, and phase‐randomized: orange) and FSC plots of the model versus the final map (red) are superimposed. (c) Angular distribution of reconstructed particles. (d) Local resolution representation of Mod1p structure. Surface view and longitudinal section view (right).
**Figure S4:** Structure determination of Mod2p by cryo‐EM. (a) Image processing flow of 3D classification and reconstruction. (b) Fourier shell correlation (FSC) plots of the cryo‐EM map (unmasked: black, masked: blue, phase‐randomized corrected: green, and phase‐randomized: orange) and FSC plots of the model versus the final map (red) are superimposed. (c) Angular distribution of reconstructed particles. (d) Local resolution representation of Mod2p structure. Surface view and longitudinal section view (right).
**Figure S5:** Cryo‐EM densities and structural models of Mod1p and Mod2p. Selected polypeptides and cofactors. The density maps are shown at a contour level of 4.0 *σ* except for the region with the lowest sequence homology (3.0 σ). The color codes are the same as in Figure 2. Red and orange arrowhead indicate the arabityl C2′‐OH of a‐FAD and the C2′‐OH group of canonical FAD, respectively.
**Figure S6:** Sequence alignment of Mod1p, Mod2p, and other methanol oxidases forming crystalloids in peroxisomes. Sequences were aligned using ClustalW. Blue and red arrowheads indicate charged residues near the FAD isoalloxazine ring and at the dimer interface (residues 484–561), respectively. A variable residue near the FAD pyrophosphate group is marked with a filled triangle. Distinct residues in Mod1p and Mod2p are boxed in black. Structurally conserved (Cα RMSD < 1.0 Å) and divergent regions are indicated by green and red lines, respectively. The crystalloid contact region was deduced based on the AOX1 crystal packing of *K. phaffii*. Species abbreviations: C. boidinii (CANBO), O. polymorpha (PICAN), and *K. phaffii* (KOMPC). Identical and conserved residues are shown in red and yellow, respectively. Cyan and red boxes indicate conserved key regions of H12 and H16 that are assumed to be involved in crystalloid interaction. Helix number is based on the AOX1 structure (Vonck et al., 2016), where applicable.
**Figure S7:** Thermal stability of Ogataea methanolica Mod1p (closed circles) and Mod2p (open circles) assessed by (a) DSF analysis and (b) residual AOD activity after incubation at 40ºC. (a) DSF measurements (*n* = 10) were performed using 5.4 μM Mod1p and Mod2p in the presence of 5× SYPRO Orange dye in 100 mM potassium phosphate buffer (pH 7.0).A representative DSF unfolding curve is shown. (b) Data represent the mean ± SD (*n* = 3 for residual AOD activity).
**Figure S8:** Surface charge distribution at the interface and the exterior of the octamers. Interfaces of Mod1p and Mod2p homooctamers. For calculation, one cyan colored monomer shown in Figure 1 has been removed. Dashed regions indicate interfaces between subunits. Surface models on the left are color‐coded as in Figure 1. Electrostatic potential values are color‐coded from −5.0 kT/e (red) to +5.0 kT/e (blue).
**Figure S9:** Peroixsomal crystal packing inferred from the AOX1 structure of K. pastoris. (a) Part of a Mod1p octamer in the crystal lattice (space group I432; 228 × 228 × 228 Å), containing 6 octamers (Vonck et al., 2016). Each octamer interacts with four neighbors. The region boxed region is enlarged in (b). The dimeric octamers in (b) have the same orientation as in (a), whereas in (c) they are rotated by 45°, with a slab view highlighting the dimer interaction. The crystal packing is assumed to be mediated by Helix 12 (cyan) rather than Helix 16 (shown in red, highlighting acidic residues).
**Table S1:** Cryo‐EM data collection, refinement and validation.

## Data Availability

The cryo‐EM maps and atomic models generated in this study have been deposited in the EMDB and PDB with accession codes: EMD‐67739 and PDB ID 21JU for Mod1p, EMD‐67740 and PDB ID 21JV for Mod2p. All other data are available from the authors upon reasonable request.
