# Supplementary material for: Cryo‐EM Structures of Alcohol Oxidase Isozymes Reveal Structural Determinants of Cofactor Variation and Enzymatic Activity in Ogataea methanolica
Source: Microb Biotechnol. 2026 Apr 18;19(4):e70355. doi: 10.1111/1751-7915.70355 (PMC13091009; doi:10.1111/1751-7915.70355)
Supplement: Supplementary file 1 — Figure S1: SDS‐PAGE of purified Mod1p and Mod2p. A single band of Mod1p and Mod2p showing the size of around 67 kDa. Figure S2: Cryo‐EM micrographs of the Mod1p and Mod2p octamer. A representative cryo‐EM (a) and (c) micrograph of purified Mod1p and Mod2p particles, respectively. (b) and (d) indicate representative 2D class averages from cryo‐EM micrographs of Mod1p and Mod2p, respectively. Figure S3: Structure determination of Mod1p by cryo‐EM. (a) Image processing flow of 3D classification and reconstruction. (b) Fourier shell correlation (FSC) plots of the cryo‐EM map (unmasked: black, masked: blue, phase‐randomized corrected: green, and phase‐randomized: orange) and FSC plots of the model versus the final map (red) are superimposed. (c) Angular distribution of reconstructed particles. (d) Local resolution representation of Mod1p structure. Surface view and longitudinal section view (right). Figure S4: Structure determination of Mod2p by cryo‐EM. (a) Image processing flow of 3D classification and reconstruction. (b) Fourier shell correlation (FSC) plots of the cryo‐EM map (unmasked: black, masked: blue, phase‐randomized corrected: green, and phase‐randomized: orange) and FSC plots of the model versus the final map (red) are superimposed. (c) Angular distribution of reconstructed particles. (d) Local resolution representation of Mod2p structure. Surface view and longitudinal section view (right). Figure S5: Cryo‐EM densities and structural models of Mod1p and Mod2p. Selected polypeptides and cofactors. The density maps are shown at a contour level of 4.0 σ except for the region with the lowest sequence homology (3.0 σ). The color codes are the same as in Figure 2. Red and orange arrowhead indicate the arabityl C2′‐OH of a‐FAD and the C2′‐OH group of canonical FAD, respectively. Figure S6: Sequence alignment of Mod1p, Mod2p, and other methanol oxidases forming crystalloids in peroxisomes. Sequences were aligned using ClustalW. Blue and red arrowheads indicate charg [file MBT2-19-e70355-s001.pptx]

## Slide 1
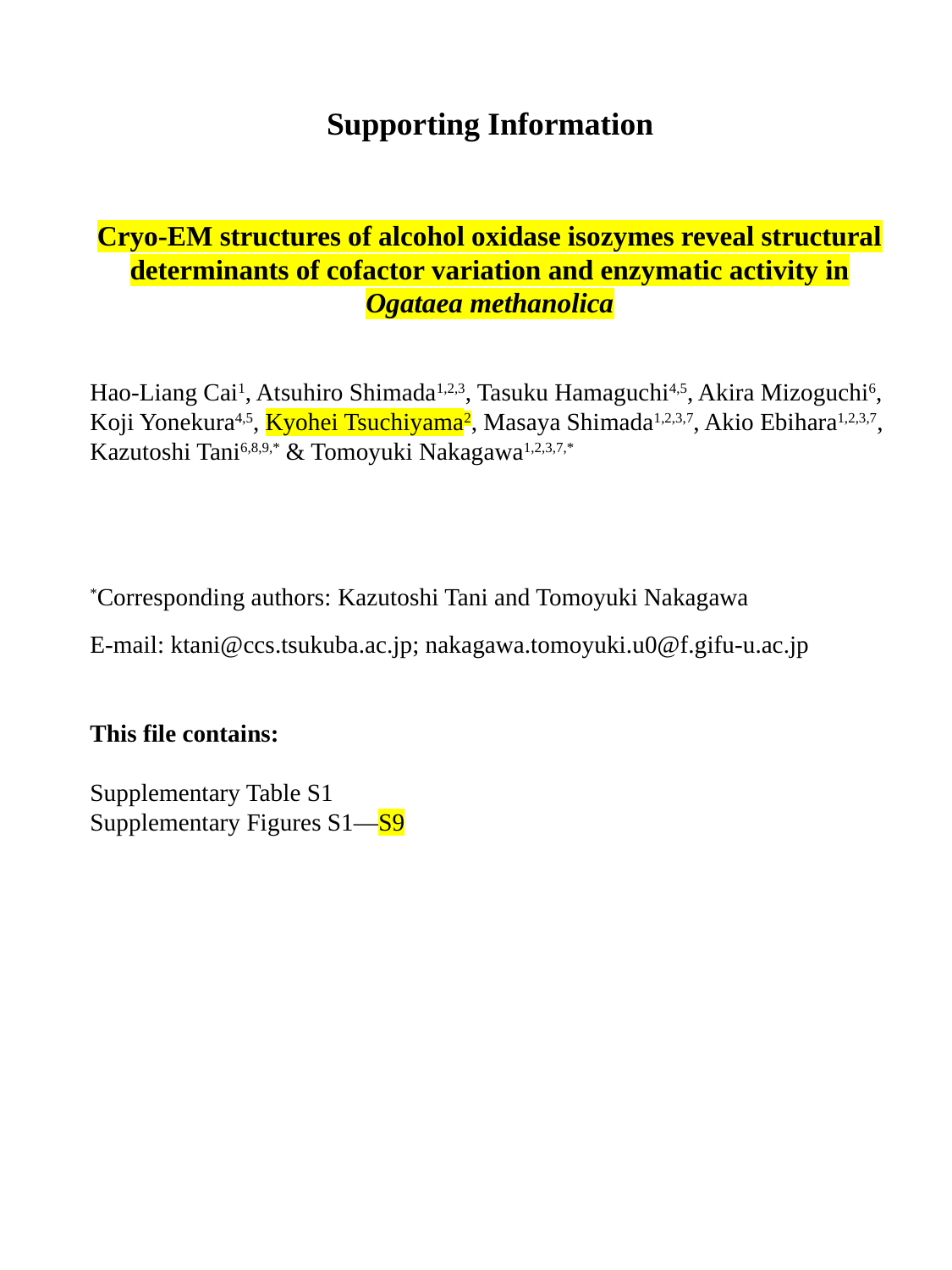

Supporting Information
Cryo-EM structures of alcohol oxidase isozymes reveal structural determinants of cofactor variation and enzymatic activity in Ogataea methanolica
Hao-Liang Cai1, Atsuhiro Shimada1,2,3, Tasuku Hamaguchi4,5, Akira Mizoguchi6, Koji Yonekura4,5, Kyohei Tsuchiyama2, Masaya Shimada1,2,3,7, Akio Ebihara1,2,3,7, Kazutoshi Tani6,8,9,* & Tomoyuki Nakagawa1,2,3,7,*
*Corresponding authors: Kazutoshi Tani and Tomoyuki Nakagawa
E-mail: ktani@ccs.tsukuba.ac.jp; nakagawa.tomoyuki.u0@f.gifu-u.ac.jp
This file contains:
Supplementary Table S1
Supplementary Figures S1—S9

## Slide 2
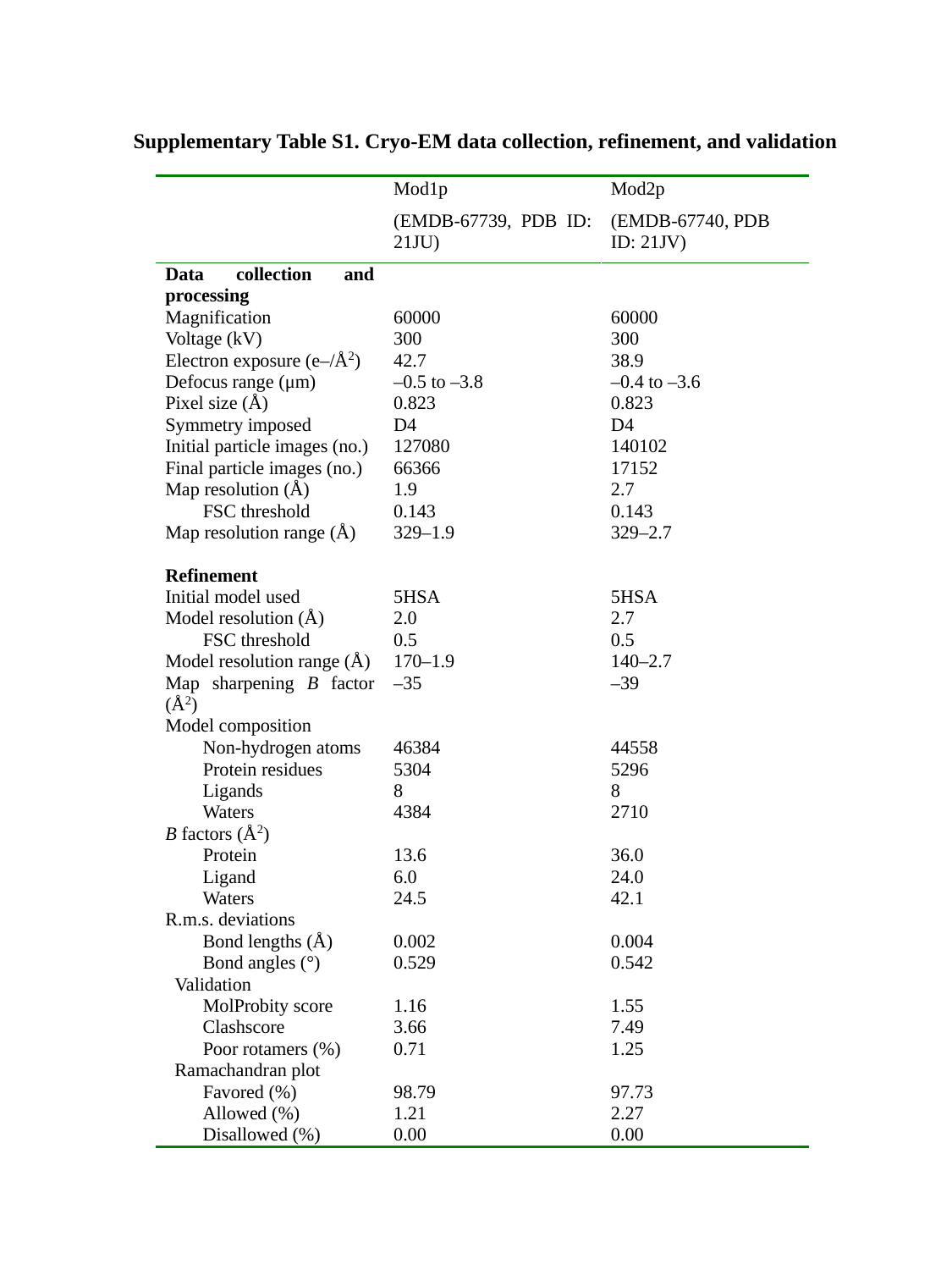

Supplementary Table S1. Cryo-EM data collection, refinement, and validation

## Slide 3
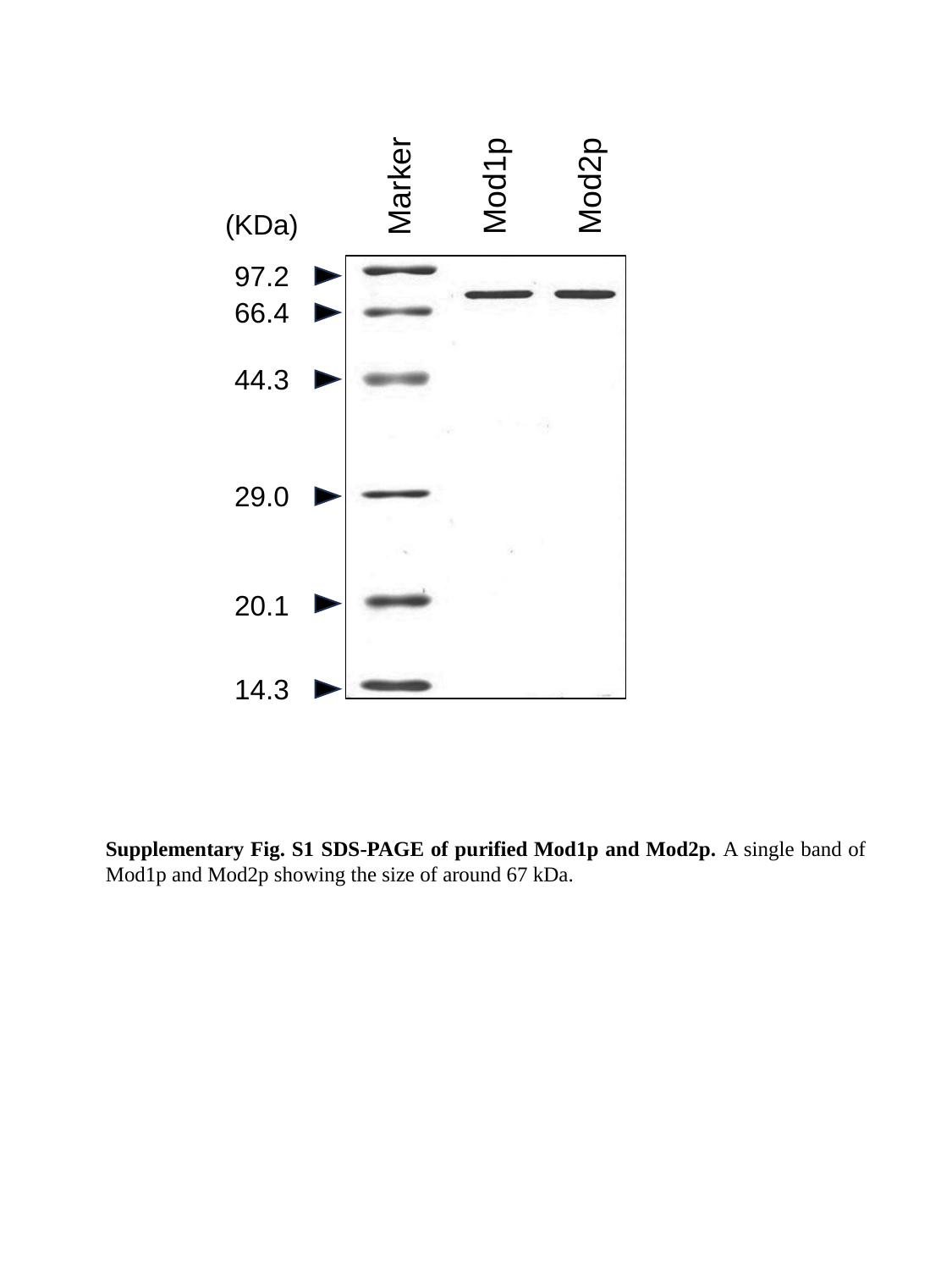

Marker
Mod1p
Mod2p
(KDa)
97.2
66.4
44.3
29.0
20.1
14.3
Supplementary Fig. S1 SDS-PAGE of purified Mod1p and Mod2p. A single band of Mod1p and Mod2p showing the size of around 67 kDa.

## Slide 4
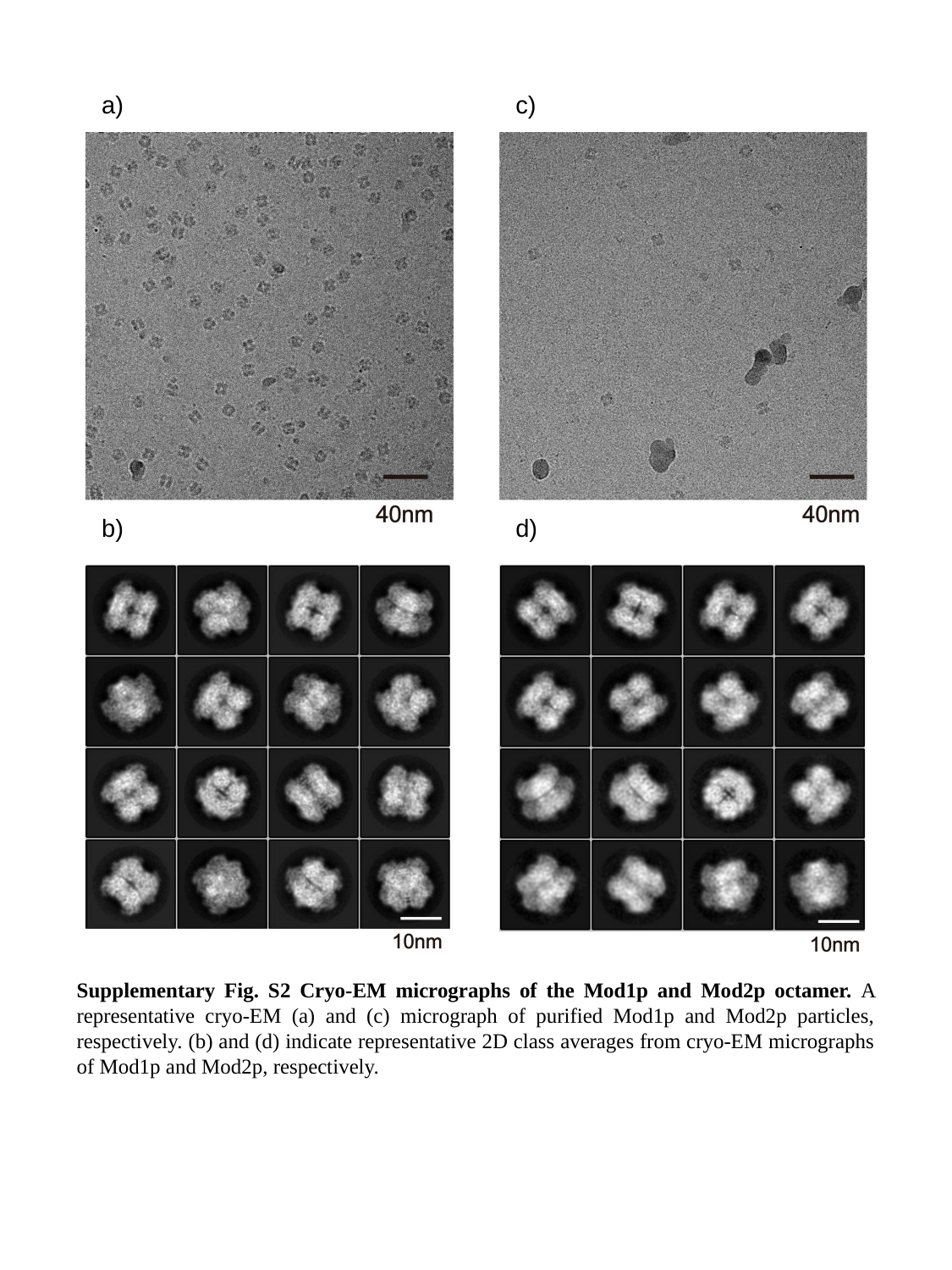

a)
c)
b)
d)
Supplementary Fig. S2 Cryo-EM micrographs of the Mod1p and Mod2p octamer. A representative cryo-EM (a) and (c) micrograph of purified Mod1p and Mod2p particles, respectively. (b) and (d) indicate representative 2D class averages from cryo-EM micrographs of Mod1p and Mod2p, respectively.

## Slide 5
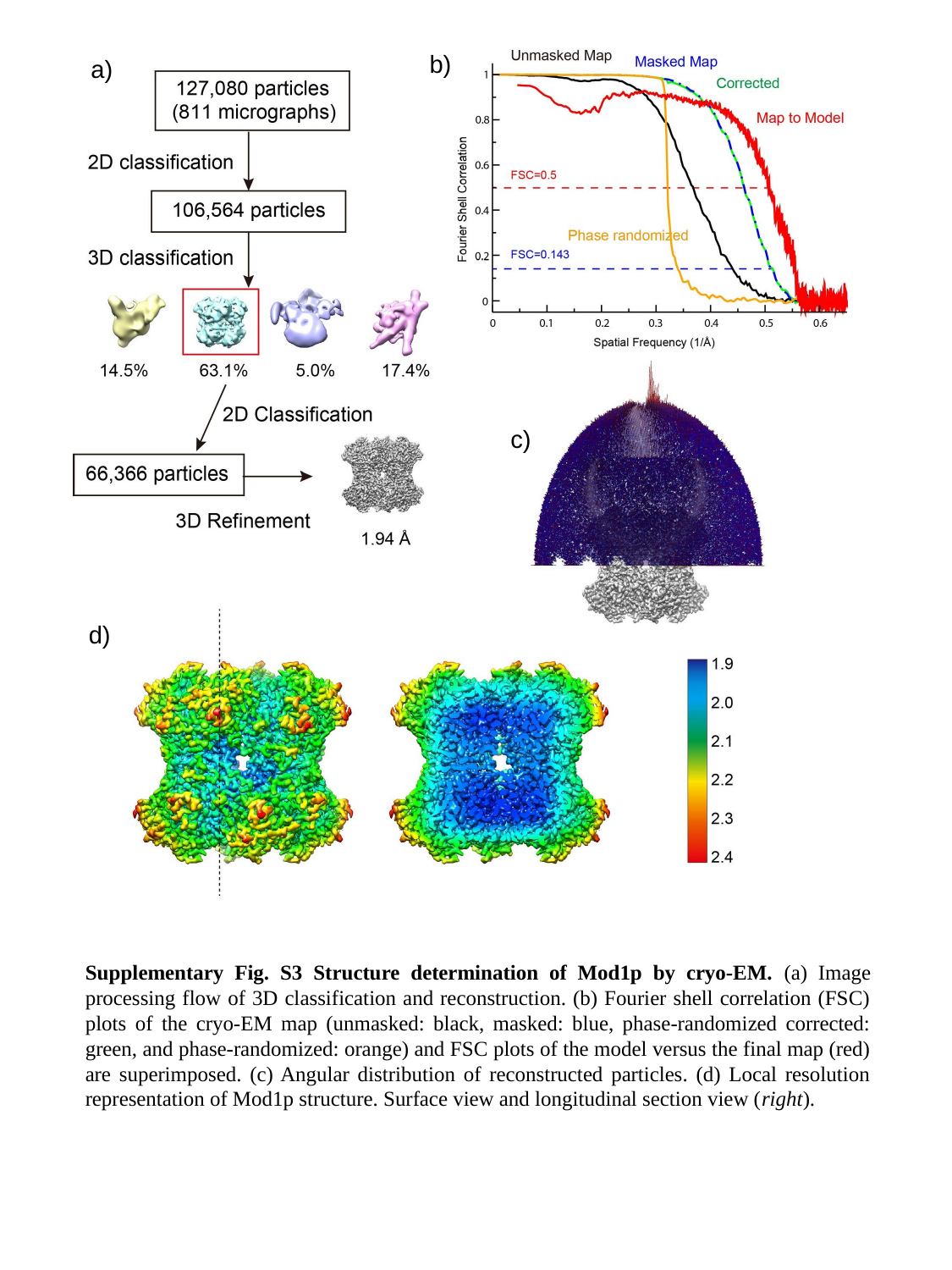

b)
a)
c)
d)
Supplementary Fig. S3 Structure determination of Mod1p by cryo-EM. (a) Image processing flow of 3D classification and reconstruction. (b) Fourier shell correlation (FSC) plots of the cryo-EM map (unmasked: black, masked: blue, phase-randomized corrected: green, and phase-randomized: orange) and FSC plots of the model versus the final map (red) are superimposed. (c) Angular distribution of reconstructed particles. (d) Local resolution representation of Mod1p structure. Surface view and longitudinal section view (right).

## Slide 6
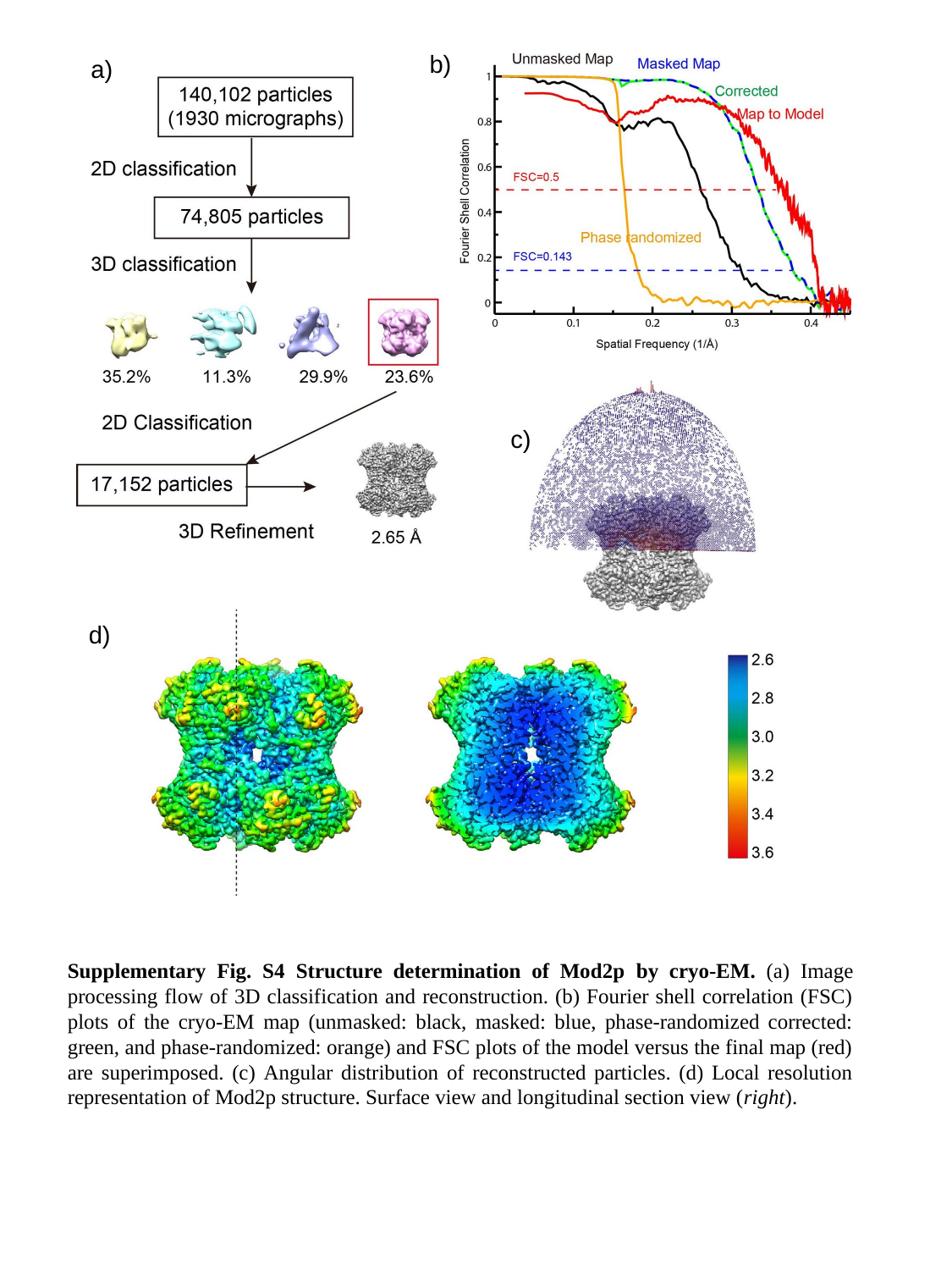

b)
a)
c)
d)
Supplementary Fig. S4 Structure determination of Mod2p by cryo-EM. (a) Image processing flow of 3D classification and reconstruction. (b) Fourier shell correlation (FSC) plots of the cryo-EM map (unmasked: black, masked: blue, phase-randomized corrected: green, and phase-randomized: orange) and FSC plots of the model versus the final map (red) are superimposed. (c) Angular distribution of reconstructed particles. (d) Local resolution representation of Mod2p structure. Surface view and longitudinal section view (right).

## Slide 7
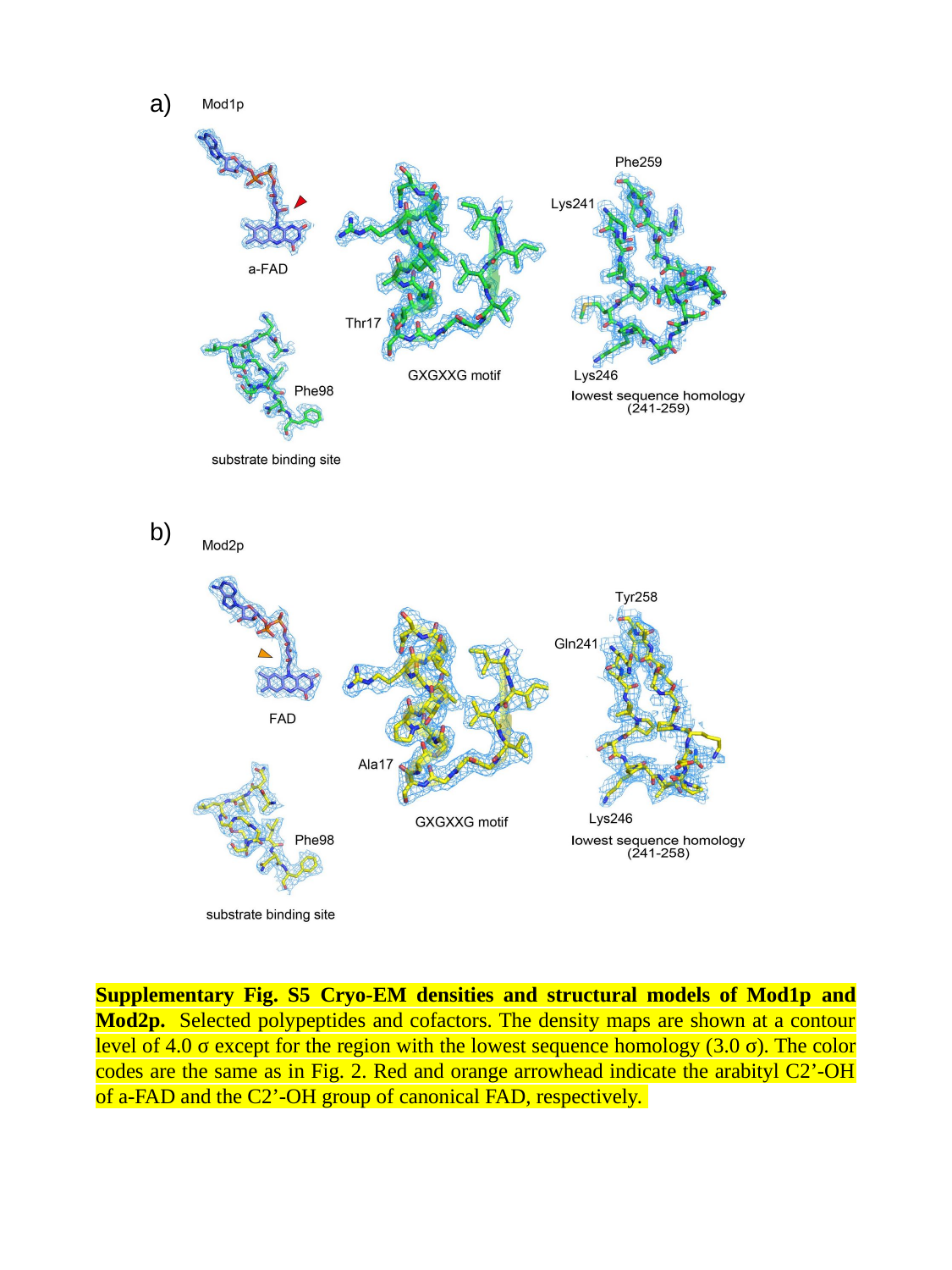

a)
b)
Supplementary Fig. S5 Cryo-EM densities and structural models of Mod1p and Mod2p. Selected polypeptides and cofactors. The density maps are shown at a contour level of 4.0 σ except for the region with the lowest sequence homology (3.0 σ). The color codes are the same as in Fig. 2. Red and orange arrowhead indicate the arabityl C2’-OH of a-FAD and the C2’-OH group of canonical FAD, respectively.

## Slide 8
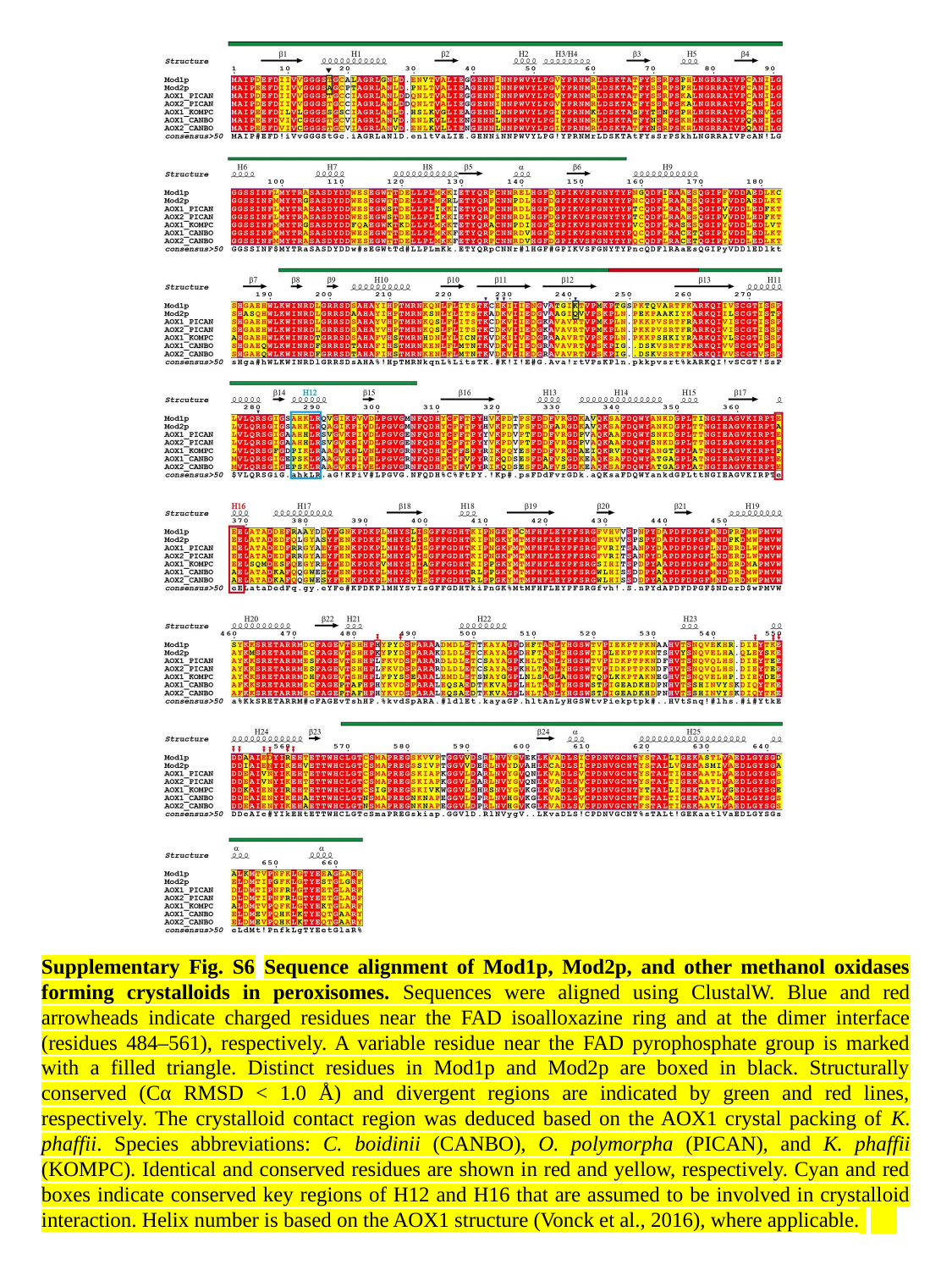

Supplementary Fig. S6 Sequence alignment of Mod1p, Mod2p, and other methanol oxidases forming crystalloids in peroxisomes. Sequences were aligned using ClustalW. Blue and red arrowheads indicate charged residues near the FAD isoalloxazine ring and at the dimer interface (residues 484–561), respectively. A variable residue near the FAD pyrophosphate group is marked with a filled triangle. Distinct residues in Mod1p and Mod2p are boxed in black. Structurally conserved (Cα RMSD < 1.0 Å) and divergent regions are indicated by green and red lines, respectively. The crystalloid contact region was deduced based on the AOX1 crystal packing of K. phaffii. Species abbreviations: C. boidinii (CANBO), O. polymorpha (PICAN), and K. phaffii (KOMPC). Identical and conserved residues are shown in red and yellow, respectively. Cyan and red boxes indicate conserved key regions of H12 and H16 that are assumed to be involved in crystalloid interaction. Helix number is based on the AOX1 structure (Vonck et al., 2016), where applicable.

## Slide 9
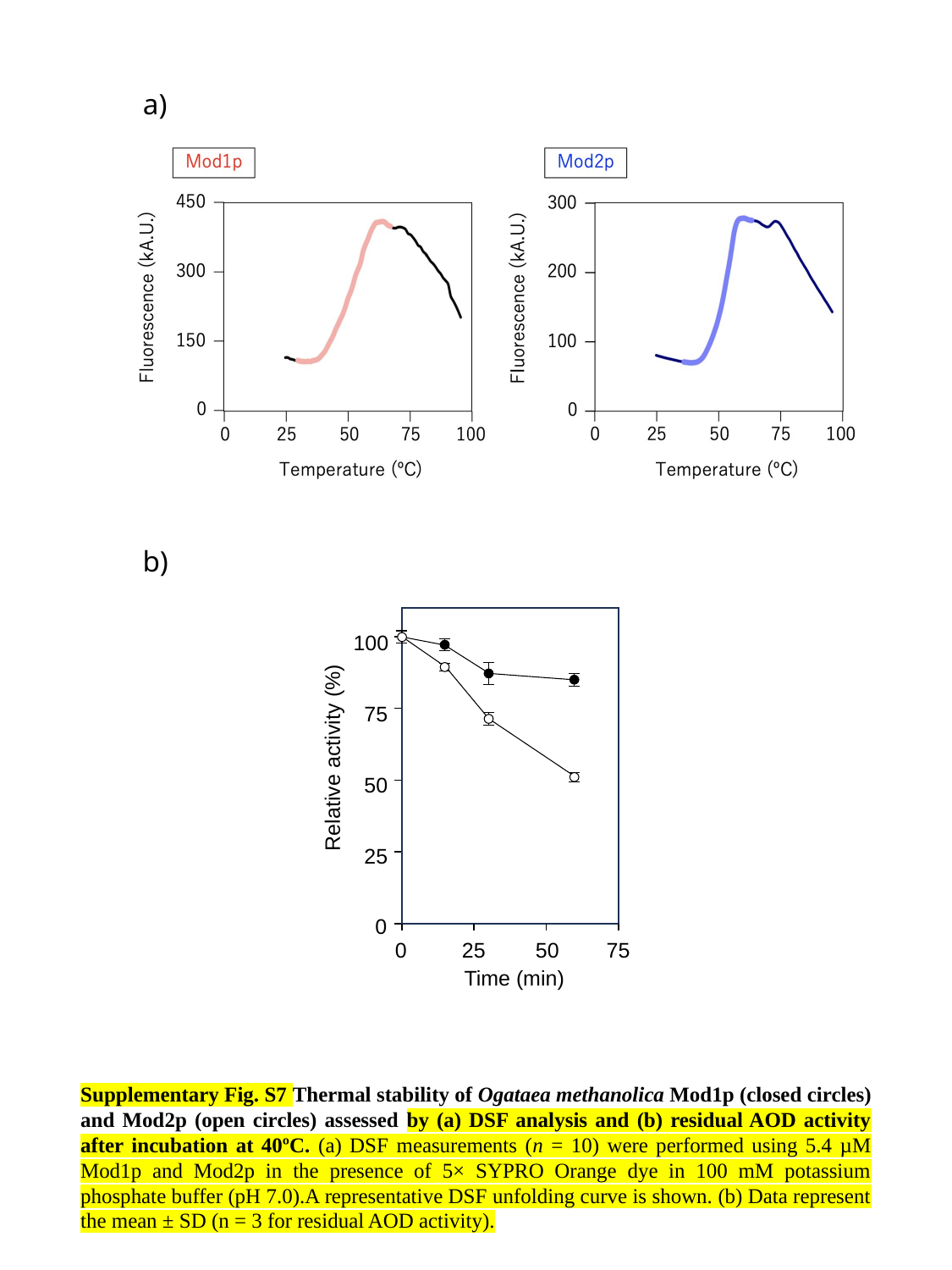

a)
b)
100
75
Relative activity (%)
50
25
0
75
50
25
0
Time (min)
Supplementary Fig. S7 Thermal stability of Ogataea methanolica Mod1p (closed circles) and Mod2p (open circles) assessed by (a) DSF analysis and (b) residual AOD activity after incubation at 40ºC. (a) DSF measurements (n = 10) were performed using 5.4 µM Mod1p and Mod2p in the presence of 5× SYPRO Orange dye in 100 mM potassium phosphate buffer (pH 7.0).A representative DSF unfolding curve is shown. (b) Data represent the mean ± SD (n = 3 for residual AOD activity).

## Slide 10
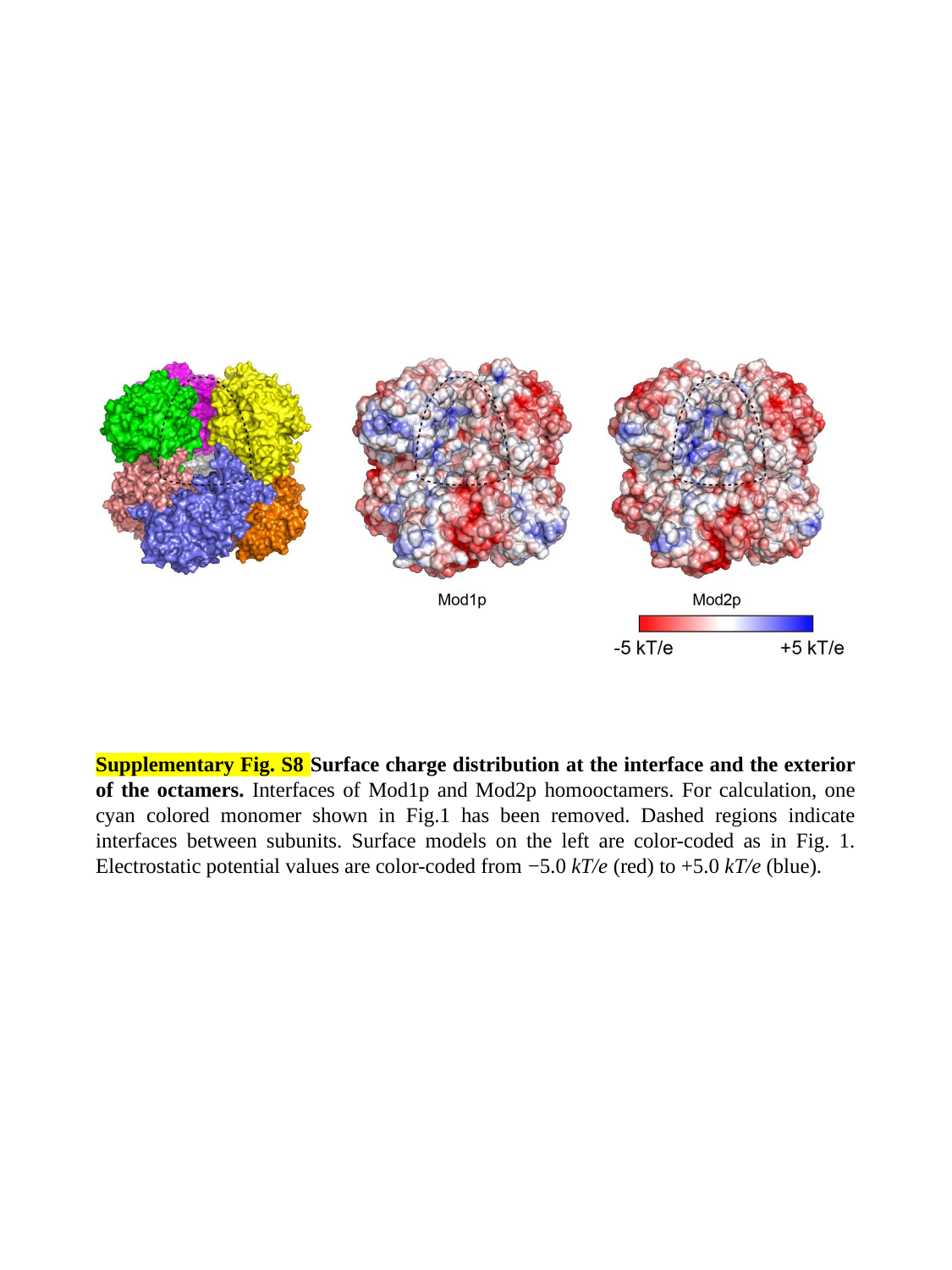

Supplementary Fig. S8 Surface charge distribution at the interface and the exterior of the octamers. Interfaces of Mod1p and Mod2p homooctamers. For calculation, one cyan colored monomer shown in Fig.1 has been removed. Dashed regions indicate interfaces between subunits. Surface models on the left are color-coded as in Fig. 1. Electrostatic potential values are color-coded from −5.0 kT/e (red) to +5.0 kT/e (blue).

## Slide 11
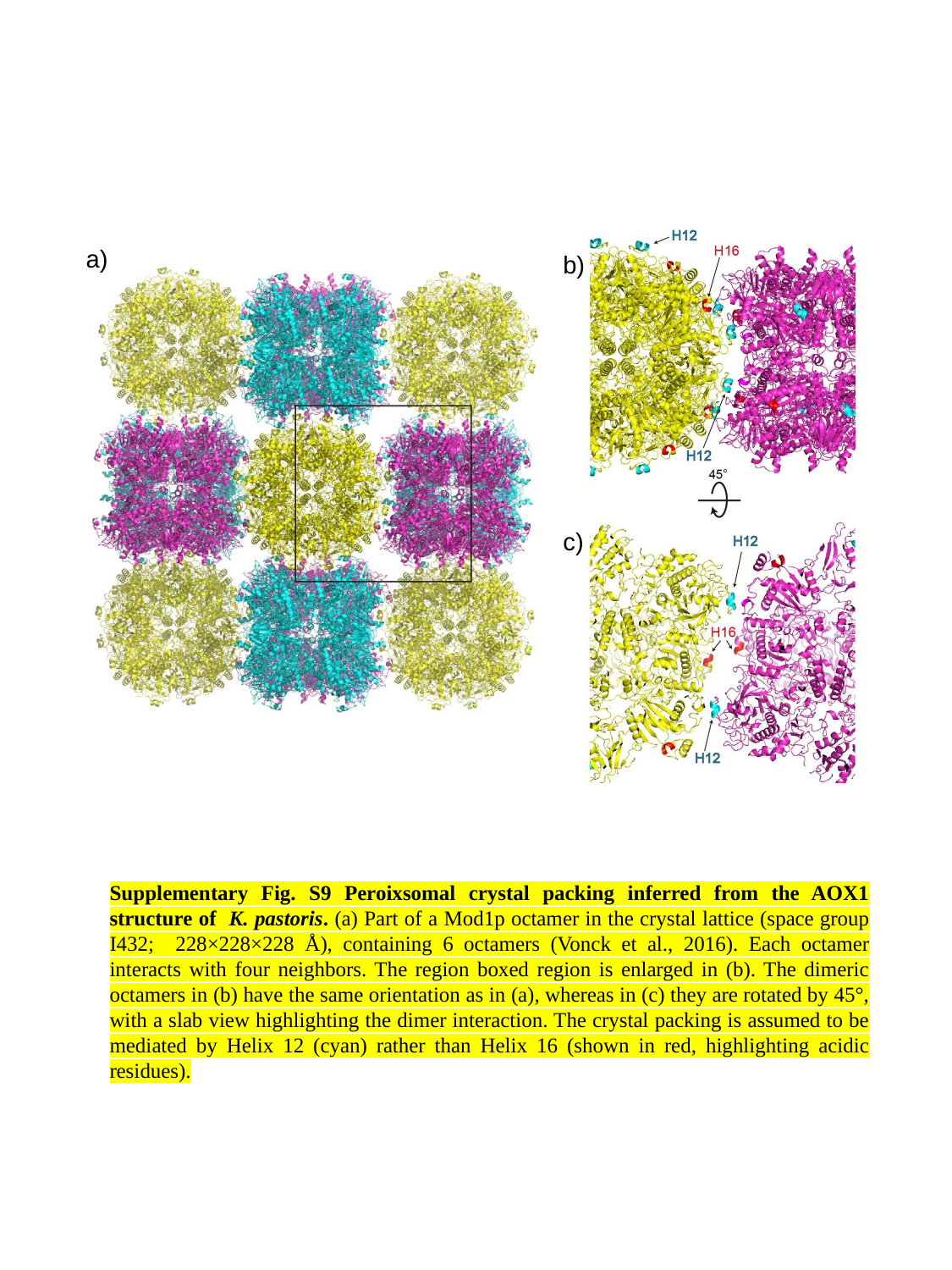

a)
b)
c)
Supplementary Fig. S9 Peroixsomal crystal packing inferred from the AOX1 structure of K. pastoris. (a) Part of a Mod1p octamer in the crystal lattice (space group I432; 228×228×228 Å), containing 6 octamers (Vonck et al., 2016). Each octamer interacts with four neighbors. The region boxed region is enlarged in (b). The dimeric octamers in (b) have the same orientation as in (a), whereas in (c) they are rotated by 45°, with a slab view highlighting the dimer interaction. The crystal packing is assumed to be mediated by Helix 12 (cyan) rather than Helix 16 (shown in red, highlighting acidic residues).
